# Learning from Nature: From a Marine Natural Product to Synthetic Cyclooxygenase‐1 Inhibitors by Automated De Novo Design

**DOI:** 10.1002/advs.202100832

**Published:** 2021-06-27

**Authors:** Lukas Friedrich, Gino Cingolani, Ying‐Hui Ko, Mariaclara Iaselli, Morena Miciaccia, Maria Grazia Perrone, Konstantin Neukirch, Veronika Bobinger, Daniel Merk, Robert Klaus Hofstetter, Oliver Werz, Andreas Koeberle, Antonio Scilimati, Gisbert Schneider

**Affiliations:** ^1^ Department of Chemistry and Applied Biosciences ETH Zurich Vladimir‐Prelog‐Weg 4 Zurich 8093 Switzerland; ^2^ Department of Biochemistry and Molecular Biology Sidney Kimmel Cancer Center Thomas Jefferson University 1020 Locust Street Philadelphia PA 19107 USA; ^3^ Department of Pharmacy – Pharmaceutical Sciences University of Bari Via E. Orabona 4 Bari 70125 Italy; ^4^ Michael Popp Institute and Center for Molecular Biosciences Innsbruck (CMBI) University of Innsbruck Innsbruck 6020 Austria; ^5^ Institute of Pharmaceutical Chemistry Goethe‐University Max‐von‐Laue Straße 9 Frankfurt am Main 60438 Germany; ^6^ Department of Pharmaceutical/Medicinal Chemistry Friedrich‐Schiller‐University Jena Philosophenweg 14 Jena 07743 Germany; ^7^ ETH Singapore SEC Ltd 1 CREATE Way, #06‐01 CREATE Tower Singapore 138602 Singapore

**Keywords:** chemoinformatics, computational chemistry, drug design, machine learning, natural product

## Abstract

The repertoire of natural products offers tremendous opportunities for chemical biology and drug discovery. Natural product‐inspired synthetic molecules represent an ecologically and economically sustainable alternative to the direct utilization of natural products. De novo design with machine intelligence bridges the gap between the worlds of bioactive natural products and synthetic molecules. On employing the compound Marinopyrrole A from marine *Streptomyces* as a design template, the algorithm constructs innovative small molecules that can be synthesized in three steps, following the computationally suggested synthesis route. Computational activity prediction reveals cyclooxygenase (COX) as a putative target of both Marinopyrrole A and the de novo designs. The molecular designs are experimentally confirmed as selective COX‐1 inhibitors with nanomolar potency. X‐ray structure analysis reveals the binding of the most selective compound to COX‐1. This molecular design approach provides a blueprint for natural product‐inspired hit and lead identification for drug discovery with machine intelligence.

## Graphical abstract

Machine learning models identify cyclooxygenase (COX) activity of the anticancer marine natural product Marinopyrrole A, and automatically construct potent new COX‐1 inhibitors, taking only the natural product as a design template. Crystallographic analysis reveals a unique binding mode of the computer‐generated COX‐1 inhibitor. This approach can provide a template for future drug design in low‐data scenarios.

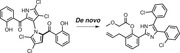

## 1 Introduction

**Figure 1:**
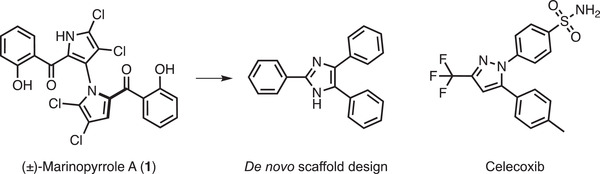
Natural product‐inspired scaffold hopping. Chemical structure of (±)‐Marinopyrrole A (**1**, left), which served as the design template, and the most prominent de novo generated molecular scaffold (2,4,5‐triphenyl imidazole, Iophine, middle). The structure of the non‐steroidal anti‐inflammatory drug celecoxib is shown for comparison (right).

Natural products are an important source of inspiration for medicinal chemists. Reportedly, more than one third of all drugs approved by the U.S. Food and Drug Administration are natural products or natural product‐inspired drugs.^[^ ^1^ ^]^ Natural products and their chemical building blocks are also preferred starting points for small‐molecule drug discovery.^[^ ^2^ ^]^ However, the biological activity of most natural products is unknown, and many pharmacologically active natural products are scarce, precluding their reaping from natural sources or requiring elaborate synthetic routes, which renders industrial production unattractive.^[^ ^3^ ^]^ Consequently, the full potential of natural products for drug discovery remains mostly untapped and unexplored. Herein, we present an efficient computational strategy for target identification and de novo design of synthetically accessible natural product mimetics. This integrated approach combines automated, rule‐based molecule construction with machine learning and experimental validation in a rapid design‐make‐test‐analyze cycle. For experimental proof of concept, we selected marine natural product Marinopyrrole A (compound **1**, **Figure** **1**) as the design template for the automated generation of new molecules.^[^ ^4^ ^]^ This compound possesses not only anti‐bacterial properties but also potent anticancer activity.^[^ ^5^, ^6^ ^]^ The shortest known total synthetic route afforded (±)‐Marinopyrrole A in five steps and 16% overall yield.^[^ ^7^ ^]^ Thus, the primary design goal was to computationally obtain novel, more easily synthesizable druglike molecules that share disease‐relevant macromolecular targets with Marinopyrrole A.

In addition to obtaining novel natural product‐inspired synthetic compounds, in this study, we aimed to analyze the degree to which the bioactivities of the design template are transferred to the de novo generated molecules. These designs should be (iso)functional rather than structural mimetics of the natural product template (“new structure, same function”). For computational structure generation, the DOGS (design of genuine structures) de novo design algorithm was utilized.^[^ ^8^ ^]^ The CATS (chemically advanced template search) distance metric was employed for ranking the computer‐generated molecules.^[^ ^9^ ^]^ The DOGS method constructs new molecules by combining molecular building blocks according to a list of in silico chemical transformations.^[^ ^10^ ^]^ Importantly, this molecular design algorithm is solely guided by the molecular similarity between the template and virtual molecules and does not rely on activity prediction for molecule construction and selection. In contrast to rule‐free generative machine learning models,^[^ ^11^, ^12^ ^]^ the algorithm generates molecules in a forward‐synthetic fashion and is thus able to suggest synthetic routes for the designs. The results of this study suggest that this approach may serve as a prototype for sustainable, natural product‐inspired hit and lead identification in chemical biology and drug discovery.

## 2 Results

### 2.1 Designing the Molecules

New molecules were constructed with DOGS software from 200 randomly selected starting fragments, with a construction set of 25 563 commercially available building blocks and 58 reaction schemes.^[^ ^13^ ^]^ The enormous number of potential reaction products does not permit exhaustive enumeration of all possible virtual products. Therefore, the DOGS algorithm performed a deterministic breadth‐first search among the virtually constructed molecules that require no more than three linear synthesis steps. Otherwise, the structure generation process was unconstrained. The pairwise molecular graph similarity between generated molecules and the Marinopyrrole A template (compound **1**) served as the fitness function during the molecule construction process.^[^ ^14^ ^]^ This permissive (“fuzzy”) similarity criterion was previously shown to enable molecular scaffold hopping between the design template and generated molecules, identifying pairs of structurally dissimilar yet functionally related compounds.^[^ ^15^, ^16^, ^17^ ^]^ Other than the chemical constitution of Marinopyrrole A, no other information (e.g., 3D conformation, target information) was used in the molecular design process.

**Figure 2:**
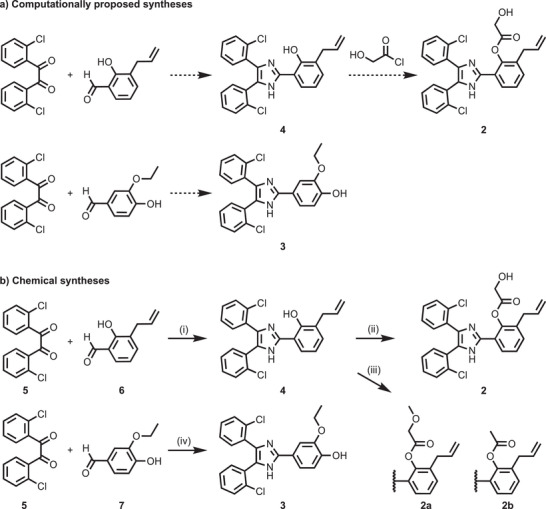
Synthetic routes to de novo designs **2** and **3**. Molecule construction, as suggested by a) the molecular design algorithm and b) the actual chemical synthesis. Both routes consist of an imidazole formation via Debus–Radziszewski reaction. To synthesize compound **2** from the imidazole intermediate **4**, esterification of the phenol group with 2‐hydroxyacetyl chloride was proposed by the software. b) Chemical synthesis of compounds **2**, **2a**, **2b**, and **3**. Reagents and conditions: i) NH_4_OAc, AcOH, reflux, 5 h, 66%; ii) (I) TBDPS‐protected glycolic acid, DCC, DMAP, CH_2_Cl_2_, RT, 16 h, 38%; iii) TBAF, AcOH, THF, 0 °C to RT, 2 h, 82% (**2**); iv) 2‐methoxyacetic acid or acetic acid, DCC, DMAP, CH_2_Cl_2_, RT, 16 h, 57% (**2a**), 53% (**2b**); iv) NH_4_OAc, AcOH, µw irradiation, 180 °C, 5 min, 48%.

For the Marinopyrrole A template, the DOGS algorithm generated a total of 802 de novo designs, comprising 334 unique molecular scaffolds (Figure S1, Supporting Information).^[^ ^18^ ^]^ The designs were ranked according to their topological pharmacophore similarity to Marinopyrrole A (CATS distance metric; lower distance values indicate more similar compounds in terms of the CATS molecular representation).^[^ ^9^ ^]^ The 100 top‐ranking designs presented CATS distances < 1.8 (Table S1, Supporting Information), suggesting a balanced compromise between conservative (small distance) and explorative (large distance) scaffold variations. This set of molecules contained 38 unique scaffolds, among which 2,4,5‐triphenyl imidazole (lophine)^[^ ^19^ ^]^ was the most frequent (34%, 15% of all 802 generated molecules, Figure 1; Figure S2, Supporting Information). Among designs containing this scaffold (Figure S3, Supporting Information), compounds **2** (best ranking, CATS distance = 1.45) and **3** (CATS distance = 1.70) were selected for synthesis. For these two de novo designs, the DOGS algorithm suggested strikingly similar synthetic routes (**Figure** **2**a).

### 2.2 Synthesizing the Molecules

The computationally proposed synthetic procedures involve imidazole formation from a dicarbonyl compound, an aldehyde, and ammonia, known as the Debus–Radziszewski reaction.^[^ ^20^ ^]^ To obtain compound **2** from intermediate product **4** of this reaction, the DOGS software proposed esterification of the phenol with an acyl halide. Imidazole synthesis was conducted for compounds **4** and **3** as proposed by the software in moderate yields (66% and 48%, respectively). Cyclization of compound **4** was successful under conventional oil bath heating, whereas compound **3** was prepared under microwave irradiation in a sealed vial. To prevent the self‐reaction of glycoloyl chloride in the computationally proposed esterification approach, this second synthetic step for compound **2** was achieved by Steglich esterification^[^ ^21^ ^]^ using *N*,*N*′‐dicyclohexylcarbodiimide (DCC) and 4‐dimethylaminopyridine (DMAP) as a nucleophilic catalyst, and silyl protection of glycolic acid, followed by desilylation with tetrabutylammonium fluoride (TBAF) (Figure 2b). Derivatives **2a** and **2b** were synthesized using the same synthetic strategy.

### 2.3 Testing the Biological Activity of the Designs

To identify macromolecular targets of the template Marinopyrrole A and its computationally designed mimetics, we employed SPiDER (self‐organizing map‐based prediction of drug equivalence relationships) target prediction software.^[^ ^22^ ^]^ The SPiDER algorithm infers the potential biological targets of a given query molecule from comparisons with local ensembles of similar compounds and known bioactivities. This is achieved by two cascaded machine learning models (self‐organizing maps), considering the molecular similarity in terms of physicochemical properties and molecular pharmacophore features, respectively.^[^ ^23^ ^]^ In previous studies, SPiDER was successfully applied to predict targets of natural products and small molecules.^[^ ^24^, ^25^, ^26^ ^]^ Predictions with *p* ≤ 0.05 were considered meaningful. Marinopyrrole A received the fewest target predictions (*n* = 8), with compound **3** demonstrating the highest number of predictions (*n* = 43). Furthermore, seven of the eight computed targets of Marinopyrrole A were additionally suggested for all of the mimetics (prostanoid receptors, cannabinoid receptors, peroxisome proliferator‐activated receptors (PPAR), corticotropin‐releasing hormone receptors (CRF), cyclooxygenases (COXs), serine/threonine protein kinases, and glucagon receptor; Table S2, Supporting Information).

In a preliminary screen, the compounds were tested at a concentration of 50 µm to determine activity toward selected members of the predicted target families. Compounds **2** (IC_50_ = 1.2 ± 1.2 µm, *K*_B_ = 0.6 µm), **3** (IC_50_ = 4.3 ± 1.2 µm, *K*_B_ = 2.2 µm), and **4** (IC_50_ = 1.4 ± 1.1 µm, *K*_B_ = 0.7 µm) demonstrated an antagonistic effect on the glucocorticoid receptor (Figure S4, Supporting Information). Compounds **3** (IC_50_ = 4.7 ± 1.2 µm, *K*_i_ = 1.7 µm), and **4** (IC_50_ = 40 ± 2 µm, *K*_i_ = 14 µm) showed antagonistic effects toward CRF1. Compound **3** additionally antagonized orexin receptor 1 (IC_50_ = 40 ± 1 µm, *K*_i_ = 8.4 µm) and cholecystokinin B receptor 2 (IC_50_ = 8.7 ± 4.6 µm, *K*_i_ = 1.1 µm). Marinopyrrole A and mimetics **2**, **2a**, and **4** were further investigated for their interaction with PPAR and a panel of related nuclear receptors in reporter gene assays (Figure S5, Supporting Information). Only Marinopyrrole A showed activity in these assays, namely the natural product activated PPAR*δ* (EC_50_ = 0.9 ± 1.2 µm), as predicted, as well as retinoic acid receptor *α* (EC_50_ = 0.6 ± 0.1 µm), vitamin D receptor (EC_50_ = 1.1 ± 0.1 µm), and liver X receptor *β* (EC_50_ = 0.3 ± 0.4 µm) (Figure S6, Supporting Information). The human glucocorticoid receptor, and orexin receptors 1 and 2 were additionally confirmed as known targets of Marinopyrrole A (Table S3, Supporting Information).

The preliminary assay results indicated pronounced COX‐1‐inhibitory activity of the compounds. COX‐1 was not known as a target of Marinopyrrole A. Evidently, the computer‐generated molecules inherited this activity from the natural product template. Therefore, the compounds were further investigated using cell‐free assays (**Table** **1** **;** Figures S7 and S8, Supporting Information). Concordant results were obtained independently of whether the COX product 12(*S*)‐hydroxy‐5‐*cis*‐8,10‐*trans*‐heptadecatrienoic acid (12‐HHT) was analyzed, or the enzyme's endoperoxidase activity was measured (Figure S9, Supporting Information). Marinopyrrole A inhibited COX‐1 in the double‐digit micromolar range (IC_50_ = 16.6 ± 2.3 µm), whereas compounds **2**, **2a**, and **4** were confirmed to demonstrate potent COX‐1 inhibition with nanomolar (**2**, IC_50_ = 0.10 ± 0.05 µm; **2a**, IC_50_ = 0.160 ± 0.001 µm) or micromolar (**4**, IC_50_ = 2.1 ± 0.3 µm) activity (Table 1). However, inhibition of COX‐2 was instead only evident at high concentrations of Marinopyrrole A (IC_50_ = 45 ± 21 µm) and its synthetic mimetics (**2**, IC_50_ = 12 ± 6 µm, **2a**, IC_50_ > 100 µm; **4**, IC_50_ = 47 ± 11 µm; Table 1). At the time of this study, there were 7911 COX‐1 and 9648 COX‐2 inhibitors in the ChEMBL24 database,^[^ ^27^ ^]^ of which only seven compounds, annotated as “selective COX‐2 inhibitors”,^[^ ^28^ ^]^ contained the 2,4,5‐triphenyl imidazole scaffold similar to the de novo designs, although with different substitution patterns and the non‐steroidal anti‐inflammatory drug (NSAID) celecoxib present among them (Figure 1).

**Table 1:** Effect of Marinopyrrole A (**1**) and mimetic compounds **2**, **2a**, **2b**, **3**, and **4** on isolated bovine cyclooxygenase (COX)‐1, human recombinant COX‐2, and COX‐1 activity in human platelets

	COX‐1 (enzyme)		COX‐2 (enzyme)		
Compound	Residual activity / [%]	IC_50_ / [µm]	Residual activity / [%]	IC_50_ / [µm]	COX‐1 (platelets) IC_50_ / [µm]
**1**	7.7 ± 3.9^a^ *	16.6 ± 2.3	41.6 ± 2.8^a^ *	45.2 ± 21.3	18.7 ± 1.1
**2**	1.5 ± 0.4^a^ ***	0.101 ± 0.051	26.6 ± 4.8^a^ *	11.8 ± 5.5	0.009 ± 0.000
**2a**	21.6 ± 6.3^a^ **	0.160 ± 0.001	99.2 ± 27.2^a^	n.d.	0.013 ± 0.005
**2b**	71.0 ± 2.6^b^ *	n.d. (>100)	92.4 ± 23.5^b^	n.d.	0.056 ± 0.015
**3**	22.9 ± 8.5^a^ *	29.7 ± 18.1	27.5 ± 13.8^a^ *	62.4 ± 16.8	1.9 ± 0.1
**4**	17.5 ± 5.6^a^ *	2.1 ± 0.3	34.0 ± 3.2^a^	46.6 ± 10.6	1.9 ± 0.4
Indomethacin	28.5 ± 3.5^b^ ***	2.8 ± 0.3	34.7 ± 5.2^b^ ***	10.6 ± 6.6	0.008 ± 0.002

^a,b^Residual activities (% of control) at 100 or 10 µM compound concentration, respectively; IC_50_ values are given as mean ± SEM of single determinations obtained in three or six (indomethacin) independent experiments; *p < 0.05, **p < 0.01, ***p < 0.001; paired student *t*‐test; n.d., not determined; IC_50_, half maximal inhibitory concentration.

**Figure 3:**
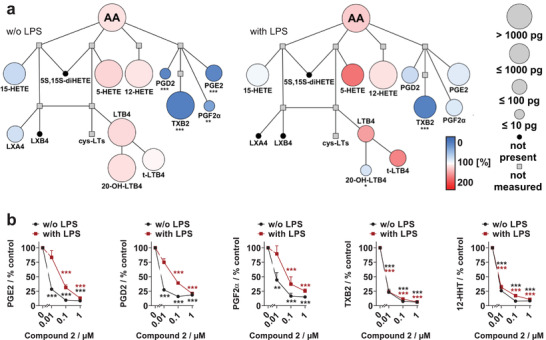
Compound **2** preferentially inhibits COX‐1 product formation in human monocytes. Monocytes were directly treated with vehicle (DMSO) or compound **2** (“w/o LPS”) or first activated with LPS to induce COX‐2 expression (“with LPS“). Then, monocytes were activated with A23187, and lipid mediator profiles were analyzed by UPLC‐MS/MS. a) Quantitative illustration of the arachidonic acid (AA)‐derived lipid mediator network of monocytes treated with compound 2 (0.01 µm) compared with vehicle control. The node size represents the average concentration in picograms (pg), and the color intensity denotes the fold change for each lipid mediator from *n* = 3 independent experiments. b) Comparison of the inhibition of COX product formation by compound **2** in A23187‐treated monocytes with and without pre‐treatment with LPS. Mean ± SEM from *n* = 3 independent experiments as a percentage of vehicle control. *p < 0.05, **p < 0.01, ***p < 0.001 versus vehicle control; repeated measures two‐way ANOVA + Tukey's post hoc test of logarithmized data. AA, arachidonic acid; (di)HETE, (di)hydroxy‐eicosatetraenoic acid; LT, leukotriene; t‐LTB_4_, trans‐LTB_4_ isomers; 20‐OH‐LTB_4_, 20‐hydroxy‐LTB_4_; LX, lipoxin; PG, prostaglandin E_2_, D_2_, F_2*α*_; TXB2, thromboxane B_2;_ 12‐HHT, 12‐hydroxyheptadecatrenoic acid; COX, cyclooxygenase; DMSO, dimethyl sulfoxide; UPLC‐MS/MS, ultra‐performance liquid chromatography‐tandem mass spectrometry.

COX‐1 dominates prostanoid formation in monocytes and platelets, with the latter exclusively expressing COX‐1 but not COX‐2. Both compound **2** (IC_50_ = 0.009 ± 0.000 µm) and its analogs **2a** (IC_50_ = 0.013 ± 0.005 µm) and **2b** (IC_50_ = 0.056 ± 0.015 µm) showed pronounced COX‐inhibitory activity in human platelets (Table 1; Figure S9, Supporting Information) and monocytes (**Figure** **3** **;** Figure S10, Supporting Information; prostaglandin E_2_: **2**, IC_50_ < 0.01 µm). COX‐1 inhibition in platelets by compound **2** (IC_50_ = 0.009 ± 0.000 µm) may be considered equipotent to indomethacin (IC_50_ = 0.008 ± 0.002 µm) (Table 1; Figures S8 and S10, Supporting Information). The compounds with free phenolic alcohol, Marinopyrrole A (**1**), and compound **4** were comparably effective in platelets and against isolated COX‐1 (Table 1). The capacity of monocytes to produce prostaglandin E_2_ strongly increased when COX‐2 expression was induced by lipopolysaccharide (LPS) treatment, while the potency of compound **2** (IC_50_ = 0.065 µm) to inhibit prostaglandin E_2_ formation decreased (Figure 3; Figure S11, Supporting Information), as expected from the superior inhibition of COX‐1 over COX‐2.

The effect of compound **2** on the monocyte lipid mediator network was investigated by targeted metabololipidomics using ultra‐performance liquid chromatography‐tandem mass spectrometry (UPLC‐MS/MS). Compound **2** inhibited the biosynthesis of the COX‐derived prostanoids prostaglandin E_2_, D_2_, F_2*α*_, and thromboxane B_2_, but did not substantially suppress 5‐lipoxygenase, 12‐lipoxygenase, and 15‐lipoxygenase product formation or fatty acid release by phospholipase A_2_ (Figure 3a,b). In monocytes pretreated with LPS, compound **2** redirected the fatty acid substrates to the 5‐lipoxygenase pathway (Figure 3), a common feature of COX inhibitors.^[^ ^29^ ^]^ Overall, the de novo designed Marinopyrrole A mimetic **2** is a potent inhibitor of COX‐1, which preferentially inhibits the biosynthesis of COX‐1‐derived products in human platelets and monocytes.

### 2.4 Analyzing the Molecular Mechanism of COX‐1 Inhibition

**Figure 4:**
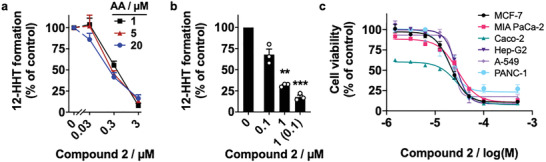
Compound **2** inhibits COX‐1 independent of the substrate concentration and kills human cancer cells. a) Effect of the substrate concentration (arachidonic acid, AA) on the inhibition of isolated COX‐1 by compound **2**. b) Reversibility of COX‐1 inhibition by compound **2**. Samples were pre‐incubated with vehicle or compound **2** for 5 min, tenfold diluted in assay buffer, and incubated for 10 min before arachidonic acid was added. Numbers in brackets indicate the diluted compound concentration after pre‐incubation. a,b) 12‐HHT was analyzed by UV‐RP‐HPLC. Mean ± SEM from *n* = 3 independent experiments as a percentage of vehicle control. **p < 0.01, ***p < 0.001 versus 20 µm arachidonic acid (a) or vehicle (b); repeated measures two‐way (a) or one‐way ANOVA (b) + Tukey's post hoc test. c) Effect of compound **2** on human cancer cell lines, as determined in a cell viability MTT assay. MCF‐7, Michigan Cancer Foundation‐7 breast cancer cells; MIA PaCa‐2, pancreatic cancer cells; Caco‐2, colon cancer cells; Hep‐G2, liver cancer cells; A‐549, adenocarcinoma alveolar basal epithelial cells; PANC‐1, pancreatic cancer cells; 12‐HHT, 12‐hydroxyheptadecatrenoic acid; COX, cyclooxygenase; UV‐RP‐HPLC, ultraviolet‐reverse phase high‐performance liquid chromatography.

**Figure 5:**
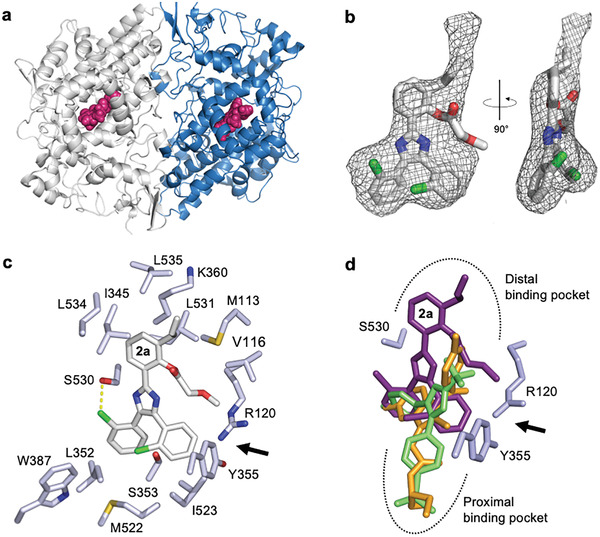
Crystallographic analysis of compound **2a** bound to *o*COX‐1. a) Cartoon representation *o*COX‐1 homodimer with the two chains colored in grey and blue, and inhibitor **2a** colored in magenta. b) Fo–Fc polder map for compound **2a** contoured at 3.2 *σ* above background. The map was calculated using all reflections between 15 and 3.35 Å resolution. The refined atomic model of compound **2a** is overlaid to electron density (mesh). c) Structural determinants for compound **2a** binding to the *o*COX‐1 active site, with residue side chains lining the *o*COX‐1 active site within 2.5–4.0 Å distance from compound **2a**. The hydrogen bridge (length = 2.9 Å) formed between catalytic Ser530 and compound **2a** is shown as a dashed line. Arg120 and Tyr355 flank the substrate entry point (black arrow) to the active site. d) Schematic of aligned COX‐1 inhibitors **2a**, (deep purple, PDB‐ID: 7JXT), indomethacin (orange, PDB‐ID: 2OYU), and celecoxib (green, PDB‐ID: 3KK6). Two opposite ligand binding pockets are indicated by dashed lines. Images and the alignment were prepared using PyMol software (Schrödinger, New York, NY, USA). *o*COX‐1, ovine cyclooxygenase‐1.

The obtained bioactivity data suggested that compound **2** inhibits COX‐1 independently of the arachidonic acid concentration (**Figure** **4**a) in a pseudo‐irreversible manner (Figure 4b). To determine the binding mode and rationalize these biochemical effects, we determined the crystal structure of the ovine COX‐1 (*o*COX‐1) in complex with the subtype‐selective derivative **2a** (*o*COX‐1 IC_50_ = 0.160 ± 0.001 µm, inactive on *h*COX‐2, Table 1). The complex was solved by molecular replacement and refined to a *R*_work/free_ of 20.97/24.99% at 3.35 Å (**Figure** **5**a; Table S4, Supporting Information, PDB‐ID: 7JXT). The overall architecture of the enzyme in this crystallographic complex was similar to previously reported structures.^[^ ^30^ ^]^ COX‐1 crystallizes as a homodimer in the asymmetric unit, consisting of two ≈72 kDa subunits tightly packed against each other via an extensive binding interface of ≈2500 Å^2^. Compound **2a** was identified in an unbiased Fo–Fc polder map within the *o*COX‐1 hydrophobic channel between residues Arg120 and Tyr355 (Figure 5b).^[^ ^31^, ^32^ ^]^ The Fo–Fc density map for compound **2a** presented clear density features at 3.2 *σ* above background, which allowed the unambiguous assignment of the ligand orientation. The methoxyacetate residue of compound **2a** was the only part of the molecule that did not have a clear density in the crystal structure (left panel of Figure 5b), possibly underscoring its flexibility and lack of binding interactions. For compound **2a**, a side view of density revealed a flat shape consistent with two planar chlorobenzenes (right panel of Figure 5b).

The crystal structure revealed that the inhibitor resided in the active site of the enzyme in an almost planar conformation. Compound **2a** bound to the substrate channel with one chlorobenzene facing Ser530, and another chlorobenzene facing down near residues Tyr355 and Arg120, the entry point to the active site. The ligand was engaged in two sets of interactions with *o*COX‐1 residues, namely a hydrogen bridge between a chlorine atom of the ligand and the hydroxyl group of residue Ser530, and multiple non‐bonded van der Waals and hydrophobic contacts with 19 *o*COX‐1 residues. The ligand portion in the distal binding pocket was surrounded by hydrophobic residues (Leu531, Leu534, Leu535, Ile345, Met113) and formed van der Waals interactions with Lys360. Furthermore, the two chlorobenzene rings were surrounded by several hydrophobic residues (Ile523, Leu352, Met522, Tyr387), suggesting van der Waals interactions with Ser353. The binding free energy of compound **2a** for *o*COX‐1 was calculated from atomic coordinates as *ΔG* = −4.1 kcal mol^−1^.

## 3 Discussion

Ligand‐based de novo molecular design was successfully combined with a machine learning model for target prediction. The rule‐based machine intelligence autonomously constructed molecules using a linear virtual synthesis approach that could be realized in practice. Notably, Marinopyrrole A received considerably fewer target predictions than the computationally generated mimetics, suggesting multiple bioactivities and greater target promiscuity of the synthetic derivatives, which is consistent with its unique chemical structure and the concept of privileged scaffolds in natural products.^[^ ^33^ ^]^ Of the eight top‐ranking predicted target families, the computer‐generated molecules were confirmed to inherit at least three targets (COX‐1, prostaglandin receptors EP1–EP3, and CRF; Table S3, Supporting Information). This result corroborates the suitability of ligand‐based similarity metrics for molecular de novo design,^[^ ^25^, ^34^ ^]^ and further validates the CATS topological pharmacophore metric as suitable for compound prioritization and scaffold hopping from natural products.^[^ ^35^ ^]^ During the study, no information regarding the macromolecular targets was used in the design or selection of the new compounds. The chemical constitution of the natural product Marinopyrrole A served as the only reference information for automated ligand‐based fragment assembly. This computational approach might, therefore, prove particularly useful in low‐data situations that are restrictive for de novo drug design with generative deep learning, thereby complementing so‐called “one‐shot” methods.^[^ ^36^ ^]^ Indeed, the concept of this rule‐based compound construction strategy perfectly complements data‐hungry deep learning methods. Furthermore, it is based on established chemical transformations that can be applied without the requirement for training data, thereby mimicking a chemist's approach to drug design.

This strategy not only succeeded in identifying a COX‐1 inhibitor but also yielded compounds that are markedly potent and highly selective over COX‐2 and other enzymes involved in lipid mediator biosynthesis, as verified in innate immune cells by metabololipidomics. COX inhibitors are among the most prominent categories of compounds in the drug market and are categorized into the class of traditional NSAIDs that inhibit both COX‐1 and COX‐2, as well as into the class of COX‐2‐specific inhibitors, referred to as coxibs.^[^ ^37^ ^]^ For example, both indomethacin and diclofenac may be considered nonselective COX inhibitors with a slight preference for COX‐1 (Table 1, IC_50_ *o*COX‐1/*h*COX‐2 = 0.26; 0.16^[^ ^38^ ^]^), whereas celecoxib is a COX‐2‐selective inhibitor (IC_50_ *o*COX‐1/*h*COX‐2 = 600^[^ ^38^ ^]^). There are only a few known selective COX‐1 inhibitors.^[^ ^39^ ^]^ Compounds **2** and **2a** demonstrated comparable activity to other COX‐1‐selective inhibitors, including SC‐560 (IC_50_ = 9 nm)^[^ ^40^ ^]^ or FR122047 (IC_50_ = 28 nm);^[^ ^41^ ^]^ however, in contrast to these known inhibitors, they presented both selectivity and pronounced activity in intact cells and platelets, respectively. Compound **2** behaved similar to indomethacin with regard to COX‐1 inhibition in platelets and showed greater COX‐1 selectivity (Table 1, IC_50_ *o*COX‐1/*h*COX‐2 = 0.009). It remains to be determined whether this potential advantage as NSAIDs also translates to an anti‐proliferative anticancer effect, which has been reported for other selective COX‐1 inhibitors.^[^ ^42^ ^]^ Similar to the natural product template, designed compound **2** was active against several human cancer cell lines (Figure 4c). In light of these new findings, the apoptosis regulator Mcl1‐independent anticancer activity of Marinopyrrole A could be reexamined.^[^ ^43^ ^]^

Except for aspirin, which covalently and irreversibly inhibits COX by acetylation of the enzymes at Ser530, all other NSAIDs bind noncovalently as either (i) rapid and time‐dependent arachidonic acid‐competitive inhibitors or (ii) time‐independent tight inhibitors that slowly form very stable complexes with COX and are highly potent.^[^ ^44^ ^]^ Compound **2a** inhibited COX‐1 independently of the arachidonic acid concentration in a highly potent manner, which was confirmed in wash‐out experiments indicating tight binding (Figure 4b). Previous studies have revealed that small‐molecule NSAIDs interact with the proximal and central inhibitor binding side of COX, which both overlap with the binding pocket for arachidonic acid.^[^ ^45^ ^]^ The majority of these drugs inhibit both COX isoforms, although with varying degrees of selectivity. In the COX‐2 subtype, substitutions of Ile434Val, His513Arg, and Ile523Val in the substrate channel constitute the only differences between COX‐2 residues and COX‐1.^[^ ^46^ ^]^ Therefore, the design of COX‐2 selective inhibitors is focused on optimizing interactions with Arg513 at the bottom of the proximal binding pocket of the enzyme.^[^ ^47^ ^]^ This structural preference is visible in the alignment of de novo designed COX‐1 selective compound **2a** with celecoxib (Figure 5d). However, the binding pose of inhibitor **2a** markedly differed from the COX‐1 binding mode of NSAIDs such as indomethacin (PDB‐ID: 2OYU)^[^ ^48^ ^]^ and celecoxib (PDB‐ID: 3KK6)^[^ ^49^ ^]^ (Figure 5d). Unlike these inhibitors, compound **2a** was bound to the distal binding pocket of the active site. All three compounds interacted with the catalytic Ser530 and structurally overlapped in this central part, whereas compound **2a** adopted an inverted orientation, occupying a hydrophobic protein pocket lined by residues Met113, Val116, Ile345, Lys360, Leu531, Leu534, and Leu535 (Figure 5c). This binding pose mimics the bound state of the endogenous substrate arachidonic acid.^[^ ^50^ ^]^ Importantly, the molecular frameworks of the de novo molecules and COX‐2 inhibitors such as celecoxib feature the 2,4,5‐triphenyl imidazole scaffold. The main difference lies in their respective substitution patterns (Figure 1). Moreover, it should be noted that the automated molecular design process generated the same generic scaffold found in known synthetic COX‐2 inhibitors by employing only the structurally unrelated natural product Marinopyrrole A as a design template. The 4,5‐biphenyl portion of the selected lophine scaffold represents a preferred solution of the “chemical machine intelligence”. This substructure motif is conserved among the top‐ranking designs. These new molecules presented here might open up new possibilities for developing COX inhibitors.

According to the World Health Organization, the global population relies on natural products for the treatment of diseases.^[^ ^51^ ^]^ However, these natural resources are endangered and limited, and for many pharmacologically active natural products, the mode of action remains elusive. Converting the often intricate chemical structures of these natural products into synthetically more easily accessible drugs is a continuing challenge. The straightforward molecular design approach presented here combined machine learning models for chemical structure generation (DOGS), ranking (CATS), and target prediction (SPiDER) for rapid access to natural‐product‐inspired synthetic compounds and suggested synthetic routes. Each of these software modules can be replaced with alternative solutions.^[^ ^52^ ^]^ For example, on losing the forward‐synthetic concept, generative “long short‐term memory networks” were successfully employed as an alternative to the rule‐based DOGS algorithm for the constructive natural‐product‐inspired design of novel nuclear hormone receptor modulators.^[^ ^53^ ^]^ Furthermore, the software modules for molecule construction and bioactivity prediction may be combined using, for example, reinforcement or transfer learning.^[^ ^54^, ^55^ ^]^ Owing to the limited number of prospective applications, which is, in part, due to limited data availability for deep learning in drug design, any judgment on the superiority of a method may be premature. Partial predictability is a fundamental challenge for rational drug discovery.^[^ ^16^, ^56^, ^57^ ^]^ Learning from natural products with machine intelligence may offer a path forward.

## 4 Experimental Section

### Computational

Prediction of potential macromolecular targets was performed with SPiDER^[^ ^26^ ^]^ software implemented as a KNIME node (version 3.2.1, KNIME, Konstanz, Germany). Molecular scaffolds and frameworks were extracted with DataWarrior (version 4.7.2, Idorsia Pharmaceuticals, Switzerland). Substructure searching with the 2,4,5‐triphenyl imidazole scaffold query was performed in ChEMBL24 (2275906 compounds, accessed 12/14/2018).^[^ ^28^ ^]^

### Chemical Synthesis and Analytics

All chemicals were purchased in highest available purity. Reagents and solvents were used without further purification unless described otherwise. All reactions were performed in oven‐dried glassware (110 °C), in absolute solvents, and under inert atmosphere (nitrogen or argon atmosphere). Microwave reactions were carried out in a Biotage Initiator 2.5 reactor. IR spectra were recorded in ethanol on a PerkinElmer Spectrum 100 FTIR spectrometer (PerkinElmer, Waltham, MA, USA), over a scan range of 600–4000 cm^−1^. UV/vis spectra were recorded in ethanol on an Agilent Cary‐60 UV/vis spectrometer (Agilent, Santa Clara, CA, USA), over a scan range of 200–800 nm. NMR spectra were recorded on a Bruker AV 400 or Bruker AV 500 spectrometer (Bruker Corporation, Billerica, MA, USA). Chemical shifts (*δ*) were reported in ppm relative to tetramethylsilane (TMS) reference and coupling constants (*J*) were reported in Hertz (Hz). High‐resolution mass spectra (HRMS) were recorded on a Bruker maXis – ESI‐Qq‐TOF‐MS (Bruker Corporation, Billerica, MA, USA). Melting points (mp) were measured on a Büchi Melting Point M 560 (Büchi, Essen, Germany). Purity of all compounds was determined by reverse phase HPLC‐MS with UV and ESI‐MS detection on a Shimadzu (Kyoto, Japan) LC‐MS 2020 system with a Nucleodur C18 HTec column (150 × 3 mm, 5 µm, 110 Å) and a linear 50–95% or 30–95% acetonitrile in water (MilliQ) gradient containing 0.1% formic acid over 16 min with a flow rate of 0.5 mL min^−1^ at 40 °C. All compounds for biological testing had a purity >95% (area under the curve for UV250 peaks).

### 2‐Allyl‐6‐(4,5‐bis(2‐chlorophenyl)‐1H‐imidazol‐2‐yl)phenol (**4**)

1,2‐bis(2‐Chlorophenyl)ethane‐1,2‐dione (**5**, 0.52 g, 1.8 mmol, 1.0 equiv.), 3‐allyl‐2‐hydroxybenzaldehyde (**6**, 0.30 g, 1.8 mmol, 1.0 equiv.), and ammonium acetate (1.5 g, 18 mmol, 10 equiv.) were dissolved in glacial acetic acid (10 mL) and heated for 5 min to 180 °C under microwave irradiation. After cooling to room temperature, the mixture was added dropwise to a cold ammonium hydroxide solution (25%, 150 mL). A yellow precipitate was filtered off and washed with cold water. The crude product was then purified by column chromatography using hexane/CH_2_Cl_2_ (6:1 + 5% MeOH) to CH_2_Cl_2_ with 5% MeOH as mobile phase to yield the title compound as colorless solid (210 mg, 27%). mp = 70 °C; ^1^H‐NMR (400 MHz, DMSO‐*d*_6_): *δ* = 13.28 (s, 1H, NH), 13.09 (s, 1H, OH), 7.85 (dd, *J* = 7.9, 1.6 Hz, 1H, CH_ar_), 7.60–7.52 (m, 1H, CH_ar_), 7.51–7.25 (m, 7H, CH_ar_), 7.15 (dd, *J* = 7.4, 1.4 Hz, 1H, CH_ar_), 6.90 (t, *J* = 7.6 Hz, 1H, CH_ar_), 6.01 (ddt, *J* = 16.7, 10.1, 6.6 Hz, 1H, CH_2_═CH), 5.12–5.00 (m, 2H, CH═CH_2_), 3.48–3.36 (m, 2H, C_ar_CH_2_); ^13^C‐NMR (101 MHz, DMSO‐*d*_6_): *δ* = 154.5, 145.1, 136.7, 134.0, 133.2, 132.5, 132.3, 131.6, 130.6, 130.3, 129.9, 129.8, 129.4, 129.3, 127.4, 127.3, 127.0, 126.1, 122.8, 119.5, 118.6, 115.6, 112.3, 33.6; HRMS (ESI): m/z 421.0869 calculated for C_23_H_19_Cl_2_N_2_O_2_^+^ ([M+H]^+^), found 421.0871.

### 2‐Allyl‐6‐(4,5‐bis(2‐chlorophenyl)‐1H‐imidazol‐2‐yl)phenyl 2‐hydroxyacetate (**2**)

2‐((*tert*‐Butyldiphenylsilyl)oxy)acetic acid (0.13 g, 0.41 mmol, 1.2 equiv.) was dissolved in CH_2_Cl_2_ (2 mL). 4‐(Dimethylamino)pyridine (6.2 mg, 0.050 mmol, 0.14 equiv.) and **4** (0.15 g, 0.36 mmol, 1.0 equiv.) were added and the mixture was cooled to 0 °C. After 15 min, *N,N’*‐dicyclohexylcarbodiimide (44 mg, 0.21 mmol, 0.58 equiv.) in CH_2_Cl_2_ (1 mL) was added slowly and the mixture was stirred for 1 h at 0 °C. The reaction mixture was allowed to warm to room temperature and stirred for further 16 h. A white precipitate was filtered off, and the filtrate was diluted with EtOAc (20 mL), washed with water (25 mL) and brine (25 mL), dried over MgSO_4_, and concentrated under reduced pressure. The crude product was purified by column chromatography using hexane/EtOAc (7:1 to 3:1) as mobile phase to yield the intermediate 2‐allyl‐6‐(4,5‐bis(2‐chlorophenyl)‐1*H*‐imidazol‐2‐yl)phenyl‐2‐((tert‐butyldiphenylsilyl)oxy) acetate as colorless solid (96 mg, 38%). ^1^H NMR (400 MHz, acetone‐*d*_6_): *δ* = 11.77 (s, 1H), 7.99 (dd, *J* = 3.0, 6.5 Hz, 1H), 7.76–7.71 (m, 4H), 7.52 (dd, *J* = 1.8, 7.5 Hz, 1H), 7.50 (d, *J* = 1.3 Hz, 1H), 7.48 (d, *J* = 1.4 Hz, 1H), 7.46 (t, *J* = 1.6 Hz, 1H), 7.44 (t, *J* = 1.0 Hz, 1H), 7.43 (t, *J* = 1.1 Hz, 1H), 7.41 (t, *J* = 1.6 Hz, 1H), 7.38–7.33 (m, 3H), 7.30 (dd, *J* = 1.8, 7.3 Hz, 1H), 7.28–7.23 (m, 2H), 7.21 (dd, *J* = 1.4, 7.4 Hz, 1H), 5.89 (ddt, *J* = 6.7, 10.1, 16.8 Hz, 1H), 5.07–4.97 (m, 2H), 4.77 (d, *J* = 9.7 Hz, 2H), 3.27 (dd, *J* = 1.5, 6.7 Hz, 2H), 1.05 (s, 9H); ^13^C NMR (101 MHz, DMSO‐*d*_6_): *δ* = 210.07, 169.84, 136.33, 135.43, 132.38, 130.52, 130.01, 129.37, 128.39, 127.57, 127.16, 34.50, 26.88; HRMS (ESI): m/z 717.2093 calculated for C_42_H_39_Cl_2_N_2_O_3_Si^+^ ([M+H]^+^), found 717.2093.

2‐Allyl‐6‐(4,5‐bis(2‐chlorophenyl)‐1*H*‐imidazol‐2‐yl)phenyl‐2‐((tert‐butyldiphenylsilyl)‐oxy) acetate (40 mg, 56 µmol, 1.0 equiv.) was dissolved in THF (1 mL) and cooled to 0 °C. Glacial acetic acid (65 µL, 1.1 mmol, 20 equiv.) was added and the mixture was stirred for 15 min at 0 °C. Tetra‐*n*‐butylammonium fluoride (1 m solution in THF, 0.15 mL, 0.15 mmol, 2.7 equiv.) was added slowly to the reaction mixture. The mixture was stirred for 30 min at 0 °C and additional 90 min at room temperature. The mixture was then quenched with saturated aqueous ammonium chloride solution (15 mL) and extracted with EtOAc (3 × 15 mL). The combined organic layers were washed with brine (30 mL), dried over MgSO_4_, filtered and concentrated under reduced pressure. The crude product was purified by column chromatography using hexane/EtOAc (4:1 to 1:1) as mobile phase to yield compound **2** as colorless solid (22 mg, 82%). mp = 138.7 °C; IR *v*_max_ 3546.9, 3061.7, 2922.5, 1741.4, 1465.2, 1191.2, 1064.9, 761.8 cm^−1^. UV/vis *λ*_max_ 210, 225, 235, 270 nm. ^1^H NMR (500 MHz, DMSO‐*d*_6_): *δ* = 12.88–12.81 (m, 1H, NH), 7.90 (ddt, *J* = 1.9, 4.2, 5.8 Hz, 1H, CH_ar_), 7.53 (d, *J* = 8.0 Hz, 1H, CH_ar_), 7.47 (ddd, *J* = 1.8, 3.8, 7.4 Hz, 1H, CH_ar_), 7.40–7.29 (m, 7H, CH_ar_), 7.29–7.24 (m, 1H, CH_ar_), 5.97–5.86 (m, 1H, CH_2_═CH), 5.52 (dq, *J* = 2.0, 4.3 Hz, 1H), 5.13 (dt, *J* = 2.1, 17.2 Hz, 1H, CH═CH_2_), 5.10–5.05 (m, 1H, CH═CH_2_), 4.41 (dd, *J* = 3.5, 4.5 Hz, 2H, C(═O)CH_2_), 3.35–3.30 (m, 2H, C_ar_CH_2_); ^13^C NMR (126 MHz, DMSO‐*d*_6_) *δ* = 171.97, 145.74, 143.00, 137.62, 136.43, 134.55, 134.40, 133.30, 132.61, 132.53, 130.65, 130.60, 130.41, 130.22, 129.98, 129.44, 127.57, 127.54, 127.38, 127.31, 126.88, 126.64, 123.65, 117.02, 60.73, 34.53; HRMS (ESI): m/z 479.0924 calculated for C_26_H_21_Cl_2_N_2_O_3_^+^ ([M+H]^+^), found 479.0920.

### 2‐Allyl‐6‐(4,5‐bis(2‐chlorophenyl)‐1H‐imidazol‐2‐yl)phenyl 2‐methoxyacetate (**2a**)

2‐Methoxyacetic acid (13 mg, 0.14 mmol, 1.0 equiv.) was dissolved in CH_2_Cl_2_ (1 mL). 4‐(Dimethylamino)pyridine (1.7 mg, 0.014 mmol, 0.10 equiv.) and **4** (60 mg, 0.14 mmol, 1.0 equiv.) were added; the mixture was cooled to 0 °C, and *N*,*N*’‐dicyclohexylcarbodiimide (44 mg, 0.21 mmol, 1.5 equiv.) was added. The mixture was stirred for 5 min at 0 °C, then allowed to warm to room temperature and stirred for 16 h. Formed precipitates were filtered off, and the filtrate was concentrated under reduced pressure. The residue was dissolved in CH_2_Cl_2_ (20 mL) and washed with aqueous hydrochloric acid solution (0.5 m, 2 × 15 mL) and saturated aqueous NaHCO_3_ solution (2 × 15 mL). The organic layer was dried over MgSO_4_ and concentrated under reduced pressure. The crude product was purified by column chromatography using hexane/EtOAc(10:1 to 3:1) as mobile phase to yield the title compound as colorless solid (40 mg, 57%). mp = 220.1 °C; IR *v*_max_ 3061.5, 2921.0, 2958.0, 2820.1, 1764.2, 1465.3, 1415.6, 1113.2, 756.8 cm^−1^. UV/vis *λ*_max_ 210, 285 nm. ^1^H‐NMR (400 MHz, DMSO‐*d*_6_): *δ* = 12.90 (s, 1H, NH), 7.91 (dd, *J* = 7.5, 2.0 Hz, 1H, CH_ar_), 7.52 (dd, *J* = 8.0, 1.3 Hz, 1H, CH_ar_), 7.47–7.26 (m, 9H, 9 × CH_ar_), 5.90 (ddt, *J* = 16.7, 10.0, 6.6 Hz, 1H, CH_2_═CH), 5.16–5.03 (m, 2H, CH═CH_2_), 4.37 (s, 2H, OCH_2_), 3.33 (d, *J* = 7.6 Hz, 2H, C_ar_CH_2_), 3.13 (s, 3H, CH_3_); ^13^C‐NMR (100 MHz, DMSO‐*d*_6_): *δ* = 168.9, 145.0, 142.3, 136.0, 134.1, 133.9, 132.9, 132.3, 132.1, 130.2, 130.0, 129.7, 129.4, 129.0, 127.1, 126.8, 126.2, 126.0, 122.9, 116.5, 69.2, 58.2, 34.0; HRMS (ESI): m/z 493.1080 calculated for C_27_H_23_Cl_2_N_2_O_3_^+^ ([M+H]^+^), found 493.1085.

### 2‐Allyl‐6‐(4,5‐bis(2‐chlorophenyl)‐1H‐imidazol‐2‐yl)phenyl Acetate (**2b**)

Glacial acetic acid (8.6 mg, 0.14 mmol, 1.0 equiv.) was dissolved in CH_2_Cl_2_ (1 mL). 4‐(Dimethylamino)pyridine (1.7 mg, 0.014 mmol, 0.1 equiv.) and **4** (60 mg, 0.14 mmol, 1.0 equiv.) were added to the reaction mixture; the mixture was cooled to 0 °C, and *N*,*N*’‐dicyclohexylcarbodiimide (44 mg, 0.21 mmol, 1.5 equiv.) was added. The mixture was stirred for 5 min at 0 °C, then allowed to warm to room temperature and stirred for 16 h. Formed precipitates were filtered off, and the filtrate was concentrated under reduced pressure. The residue was dissolved in CH_2_Cl_2_ (20 mL) and washed with aqueous hydrochloric acid solution (0.5 m, 2 × 15 mL) and saturated aqueous NaHCO_3_ solution (2 × 15 mL). The organic layer was dried over MgSO_4_ and concentrated under reduced pressure. The crude product was purified by column chromatography using hexane/ethyl acetate (10:1 to 3:1) as mobile phase to yield the title compound as colorless solid (35 mg, 53%). mp = 225 °C; ^1^H NMR (400 MHz, DMSO‐*d*_6_): *δ* = 12.83 (s, 1H, NH), 7.87 (dd, *J* = 7.3, 2.2 Hz, 1H, CH_ar_), 7.52 (dd, *J* = 7.9, 1.4 Hz, 1H, CH_ar_), 7.44–7.25 (m, 9H, 9 × CH_ar_), 5.91 (ddt, *J* = 16.8, 10.0, 6.7 Hz, 1H, CH_2_═CH), 5.16–5.04 (m, 2H, CH═CH_2_), 3.35–3.29 (m, 2H, C_ar_CH_2_), 2.26 (s, 3H, CH_3_); ^13^C‐NMR (101 MHz, DMSO‐*d*_6_): *δ* = 169.1, 145.7, 142.5, 136.1, 134.2, 133.8, 132.8, 132.1, 132.0, 130.1, 130.0, 129.7, 129.5, 128.9, 127.1, 126.8, 126.3, 126.0, 123.2, 116.4, 34.1, 21.2; HRMS (ESI): m/z 463.0975 calculated for C_26_H_21_Cl_2_N_2_O_2_^+^ ([M+H]^+^), found 463.0976.

### 4‐(4,5‐bis(2‐Chlorophenyl)‐1H‐imidazol‐2‐yl)‐2‐ethoxyphenol (**3**)

1,2‐bis(2‐Chlorophenyl)ethane‐1,2‐dione (**5**, 0.14 g, 0.50 mmol, 1.0 equiv.), 3‐ethoxy‐4‐hydroxybenzaldehyde (**7**, 83 mg, 0.50 mmol, 1.0 equiv.), and ammonium acetate (385 mg, 5.0 mmol, 10 equiv.) were dissolved in glacial acetic acid (2.5 mL) and heated for 5 min to 180 °C under microwave irradiation. After cooling to room temperature, the mixture was added dropwise to a cold ammonium hydroxide solution (25%, 50 mL). The mixture was then extracted with EtOAc (4 × 20 mL); the combined organic layers were washed with 50% (w/w) aqueous sodium bisulfite solution (6 × 30 mL). The organic layer was dried over MgSO_4_ and concentrated under reduced pressure. The crude product was purified by column chromatography using CH_2_Cl_2_ with 5% MeOH as mobile phase to yield the title compound as colorless solid (101 mg, 48%). mp = 215 °C; ^1^H‐NMR (400 MHz, DMSO‐*d*_6_): *δ* = 12.90 (s, 1H, NH), 7.91 (dd, *J* = 7.5, 2.0 Hz, 1H, CH_ar_), 7.52 (dd, *J* = 8.0, 1.3 Hz, 1H, CH_ar_), 7.47–7.26 (m, 9H, 9 × CH_ar_), 5.90 (ddt, 3*J* = 16.7, 10.0, 6.6 Hz, 1H, CH2═CH), 5.16–5.03 (m, 2H, CH═CH_2_), 4.37 (s, 2H, OCH_2_), 3.33 (d, *J* = 7.6 Hz, 2H, C_ar_CH_2_), 3.13 (s, 3H, CH_3_); ^13^C‐NMR (101 MHz, DMSO‐*d*_6_): *δ* = 147.1, 146.6, 145.9, 136.5, 134.2, 132.6, 132.1, 132.0, 131.9, 130.7, 129.6, 129.6, 129.5, 129.2, 128.6, 126.9, 126.5, 125.7, 121.6, 118.0, 115.5, 110.2, 63.7, 14.5; HRMS (ESI): m/z 425.0818 calculated for C_23_H_19_Cl_2_N_2_O_2_^+^ ([M+H]^+^), found 425.0822.

### Activity Assays of Isolated COX‐1 and COX‐2

Purified ovine *o*COX‐1 (Cayman Chemicals; 50 units) or human recombinant *h*COX‐2 (Cayman Chemicals; 20 units) in 100 mm Tris buffer pH 8, 5 mm glutathione, 5 µm hemoglobin, and 100 µm EDTA were pre‐incubated with test compounds for 5 min at 4 °C followed by 1 min at 37 °C. Then, arachidonic acid (2 µm for COX‐2 and 5 µm for COX‐1) was added, and incubations were continued for another 10 min at 37 °C. Formation of COX‐derived 12‐HHT from arachidonic acid was analyzed by UV‐RP‐HPLC.^[^ ^58^ ^]^

### COX Colorimetric Inhibitor Screening Assay

The peroxidase activity ovine *o*COX that catalyzes the reduction of the endoperoxide PGG_2_ into the corresponding alcohol (PGH_2_), which is the precursor of PGs, thromboxane, and prostacyclin, was measured. 10 µL of test compound in DMSO was added to a solution (180 µL) composed of assay buffer (0.1 m Tris‐HCl pH = 8, 160 µL), heme solution (Cayman Chemicals, Ann Arbor, MI, USA; item number: 760116 [300 µL of hemin in DMSO], 10 µL), and 10 µL of 7.49 U mL^−1^ *o*COX‐1 (Cayman item number: 760110) or 8.14 U mL^−1^ human *h*COX‐2 solution (Cayman item number: 760119), respectively. After a 5 min incubation at 25 °C, 20 µL of *N,N,N’,N’*‐tetramethyl‐*p*‐phenylenediamine (TMPD) solution (Cayman item number: 760117) and 20 µL of arachidonic acid (100 µm) were added, and the incubation was continued for 5 min at 25 °C. The appearance of oxidized TMPD was monitored by reading the absorbance at *l* = 590 nm on a Victor 3 instrument (PerkinElmer, Waltham, MA, USA). According to the manufacturer's recommendation (Cayman Chemicals), stock solutions were prepared solubilizing the test compounds in DMSO to be then diluted by the same solvent to obtain the appropriate concentrations. The concentration of DMSO in the solution was 5.3% before and 4.3% after the addition of 20 µL of TMPD solution and 20 µL of arachidonic acid.

### COX‐1 Product Formation in Human Primary Platelets

Human peripheral blood (University Hospital Jena, Germany) was obtained by venipuncture in heparinized tubes (16 IE heparin per mL of blood) with informed consent from healthy male and female adult donors (age: 18–65 years, had done 12 h fasting) that had not taken any anti‐inflammatory drugs during the previous 10 days. The registered blood donors were physically inspected by a clinician before blood collection. Experimental protocols using human platelets and monocytes were approved by the ethical commission of the Friedrich–Schiller‐University Jena, Germany. The blood was centrifuged at 4000 × *g* for 20 min at 20 °C for preparation of leukocyte concentrates, which were subjected to dextran sedimentation and centrifugation on lymphocyte separation medium (LSM 1077, GE Healthcare, Freiburg, Germany). For isolation of platelets, the supernatants were mixed with phosphate‐buffered saline (PBS) pH 5.9 (3:2), centrifuged (2100 × *g*, 15 min, 20 °C), and the pelleted platelets were resuspended in PBS pH 5.9/0.9% NaCl (1:1, v/v). Washed platelets were finally resuspended in PBS pH 7.4 containing 1 mg mL^−1^ glucose and 1 mm CaCl_2_. Freshly isolated human platelets (10^8^ mL^−1^ PBS pH 7.4 containing 1 mg mL^−1^ glucose and 1 mm CaCl_2_) were pre‐incubated with the test compounds for 15 min at 37 °C and stimulated for 10 min at 37 °C with 5 µm arachidonic acid. COX product formation was stopped after 10 min at 37 °C by addition of 1 mL ice‐cold methanol, and the formed 12‐HHT was analyzed by UV‐RP‐HPLC.^[^ ^59^ ^]^

### Lipid Mediator Profiling in Activated Human Primary Monocytes

After centrifugation of leukocyte concentrates on separation medium, as detailed for the isolation of human platelets, the peripheral blood mononuclear cell fraction was incubated in culture flasks (Greiner) for 1.5 h at 37 °C and 5% CO_2_ in RPMI 1640 medium (Sigma–Aldrich, Deisenhofen, Germany) supplemented with fetal calf serum (Sigma–Aldrich; 5%), *L*‐glutamine (Sigma–Aldrich; 2 mm), and penicillin/streptomycin (GE Healthcare; 100 U mL^−1^ and 100 µg mL^−1^). Adherent monocytes (2 × 10^6^) were harvested and either directly pre‐incubated with vehicle (DMSO) or test compounds for 10 min or first stimulated with LPS (Sigma–Aldrich, 1 µg mL^−1^) for 24 h at 37 °C and 5% CO_2_ to induce COX‐2 expression. Cells were then activated with 2.5 µm A23187 (Sigma–Aldrich) for 10 min at 37 °C. Ice‐cold methanol was added and lipid mediators were extracted by solid phase extraction (Sep‐Pak Vac 6cc 500 mg 6 mL^−1^ C18; Waters, Milford, USA) following protein precipitation at −20 °C and acidification (pH 3.5).^[^ ^60^ ^]^ d8‐5*S*‐HETE, d4‐LTB_4_, d5‐LXA_4_, d5‐RvD2, d4‐PGE_2_ (200 nm, each Cayman Chemical), and d8‐arachidonic acid (10 µm, Cayman Chemical) were used as internal standards. Eicosanoids, docosanoids, and fatty acids were separated on an ACQUITY UPLC BEH C18 column (1.7 µm, 2.1 × 100 mm; Waters) using an Acquity Ultraperformance LC system (Waters) and detected by a QTRAP 5500 mass spectrometer (Sciex) equipped with an electrospray ionization source.^[^ ^60^ ^]^ Lipid mediators were analyzed by scheduled multiple reaction monitoring in the negative ion mode. External calibration was used for quantification, with six diagnostic fragment ions and the retention time being confirmed using external standards (Cayman Chemicals).

### Reagents for Protein Crystallization

Fe^3+^‐protoporphyrin IX (heme) was purchased from Frontier Scientific (Logan, UT). *n*‐octyl *β*‐D‐glucopyranoside (*β*‐OG) and C10E6 were purchased from Anatrace (Maumee, OH). EDTA free protease inhibitor was purchased from Roche Applied Science (Penzberg, Germany). All other chemicals (reagents and solvents) were purchased from Sigma Life Science (St. Louis, MO, USA).

### Protein Expression and Purification

The gene encoding ovine COX‐1 was cloned in a modified pFastBac vector (Invitrogen, Waltham, MA, USA) engineered with an N‐terminal 8X‐His tag and a Tobacco Etch Virus protease cleavage. Generation of recombinant baculovirus, expression of recombinant his‐tagged *o*COX‐1, and purification of untagged *o*COX‐1 were carried out as described.^[^ ^31^ ^]^ Nickel‐NTA Agarose beads were purchased from Gold Biotechnology (St. Louis, MO, USA). Pure *o*COX‐1was concentrated using a 2 mL Vivaspin concentrator (Sartorius, Göttingen, Germany) to 5–6 mg mL^−1^ (as assessed by BCA protein assay, Pierce, Rockford, IL) in HEPES pH = 7.0, 40 mm NaCl, and 0.4% *β*‐OG and used for crystallization. BCA protein reagent was purchased from Pierce (Thermo Scientific, Waltham MA, USA).

### Crystallographic Methods

*o*COX‐1 was reconstituted with a twofold molar excess of heme (Fe^3+^‐protoporphyrin IX) and twofold molar excess of **2a** and allowed to incubate at room temperature for 10 min before setting up crystallization trays. Crystallization trays were set up at 25 °C using the sitting‐drop vapor diffusion method. 1 µL of protein was mixed with 1 µL of crystallization solution consisting of 0.5–0.9 m LiCl, 0.7 m sodium citrate pH 6.5, 1 mm sodium azide, and 0.3% (w/v) *β*‐OG and was equilibrated within a reservoir containing 0.5–0.9 m LiCl, 0.7 m sodium citrate pH 6.5, 1mm sodium azide, and 0.3% (w/v) *β*‐OG. Crystals appeared within 2–3 weeks. Before data collection, crystals were harvested, briefly soaked in a solution containing 1 m sodium citrate, 1 m LiCl, 0.15% *β*‐OG, and 1 mm sodium malonate as a cryo‐protectant and flash‐frozen in liquid nitrogen. Diffraction data were collected at beamline 9–2 at the Stanford Synchrotron Radiation Lightsource (SSRL, Menlo Park, CA, USA) using a Dectris PILATUS 6M detector and processed using HKL2000.^[^ ^61^ ^]^ The structure was solved by molecular replacement using the program PHASER and *o*COX‐1 (PDB 5WBE) as a search model.^[^ ^62^, ^63^ ^]^ The hexagonal asymmetric unit contains a dimer of *o*COX‐1 that was subjected to iterative cycles of reciprocal and real space refinement using distinct TLS groups as implemented in phenix.refine.^[^ ^64^ ^]^ The density for **2a** was identified in Fo‐Fc polder maps, as implemented in phenix.polder.^[^ ^65^ ^]^ Visualization and model building were done using Coot.^[^ ^66^ ^]^ The final model consisted of residues 32–584 of *o*COX‐1, two Fe3+‐protoporphyrin IX, six carbohydrates, and one compound **2a** bound in the *o*COX‐1 active site of each monomer (Table S4, Supporting Information). Figures were prepared with PyMOL (Schrödinger, New York, NY, USA).^[^ ^67^ ^]^ The binding free energy (*ΔG*) was calculated using PISA software^[^ ^68^ ^]^ and intramolecular contacts between compound **2a** and *o*COX‐1 were measured using PDBsum.^[^ ^69^ ^]^

### Preliminary Bioactivity Screening

Initial tests were conducted by Eurofins (Cerep SA, France) on a fee‐for‐service basis. The assay protocols can be found on the service provider's website at www.eurofinsdiscoveryservices.com. Initial screening measurements were conducted with two replicates at 50 µm concentration. IC_50_, *K*_i_, and *K*_B_ values were determined by the service provider from eight different concentrations with two replicates for each concentration. Log(concentration) response curves (four‐parameter logistic fit) were plotted in Prism 7 (GraphPad Software, La Jolla, CA, USA). Inhibition constants (*K*_i_) were calculated according to the Cheng–Prusoff equation (*K*_i_ = IC_50_[1+(C/*K*_D_)]^−1^), where *C* is the concentration of the radioligand in the assay and *K*_D_ the affinity of the radioligand for the receptor. Binding constants (*K*_B_) were calculated with the modified Cheng–Prussoff equation (*K*_B_ = IC_50_[1+(*C*/EC_50.C_)]^−1^), where *C* is the concentration of control binder in the assay and EC_50.C_ its EC_50_ value. Eurofins assay catalogue numbers: COX‐1 (4173), EP1 (2054), EP2 (1957), EP3 (2578), EP4 (1872), CDK1 (2875), CDK2 (2908), CDK4 (2876), SAPK2A (2881), ERK2 (2878), JNK3 (2916), GSK3beta (2879), IKKalpha (2937), IKKbeta (2938), IKKepsilon (2587), IRAK4 (2933), PKD1 (2204), ROCK2 (2884), CB1 (1744, 1745), CB2 (1746, 1747), CRF1 (505), CCK2 (1879), GR (469), OX1 (2235), OX2 (2350).

### Hybrid Reporter Gene Assays for Nuclear Receptors

The Gal4 hybrid reporter gene assays of the following nuclear receptors were conducted as described:^[^ ^70^ ^]^ peroxisome proliferator‐activated receptors *α*/*γ*/*δ* (PPAR *α*/*γ*/*δ*), liver X receptors *α*/*β* (LXR *α*/*β*), retinoic X receptor *α* (RXR*α*), retinoic acid receptor *α*(RAR*α*), farnesoid X receptor (FXR), vitamin D receptor (VDR), constitutive androstane receptor (CAR). pFA‐CMV‐based constructs comprising the ligand binding domain of the human nuclear receptor in question were used as expression plasmids for the chimera receptors. pFR‐Luc (Stratagene, La Jolla, CA, USA) served as reporter plasmid and pRL‐SV40 (Promega, Madison, WI, USA) for normalization of transfection efficiency and cell growth. The assays were conducted in 96‐well format in HEK293T cells. In brief, transient transfection was carried out using Lipofectamine LTX reagent (Invitrogen) according to the manufacturer's protocol. After transfection and incubation with test compounds (12–14 h), cells were assayed for luciferase activity using Dual‐Glo Luciferase Assay System (Promega) according to the manufacturer's protocol. Luminescence was measured with an Infinite M200 luminometer (Tecan, Männedorf, Switzerland). Normalization of transfection efficiency and cell growth was done by division of firefly luciferase data by renilla luciferase data and multiplying the value by 1000 resulting in relative light units (RLU). Fold activation was obtained by dividing the mean RLU of a test compound at a respective concentration by the mean RLU of untreated control. All hybrid assays were validated with reference agonists (PPAR*α*: GW7647; PPAR*γ*: pioglitazone; PPAR*δ*: L165041; LXR*α*/*β*: T0901317; FXR: GW4064; RXR*α*: bexarotene; RAR*α*: tretinoin; VDR: calcitriol; CAR: CITCO) which yielded EC_50_ values in agreement with literature. The assays were conducted in duplicates with at least two independent repeats and for active compounds repeated without hybrid receptor coding DNA for every test compound at the highest tested concentration to exclude unspecific effects.

### Cytotoxicity Study

All cell lines were purchased from American Type Culture Collection (ATCC; Manassas, VA, USA). Caco‐2, MIA PaCa‐2, and MCF7 cells were grown in Dulbecco's Modified Eagle's high glucose medium, Hep‐G2 cells in Minimum Essential Medium, PANC‐1 cells in RPMI‐1640 medium, in a humidified incubator at 37 °C under 5% CO_2_ atmosphere. Media were supplemented with 10% fetal bovine serum, 2 mm glutamine, 100 U mL^−1^ penicillin, and 100 µg mL^−1^ streptomycin. A‐549 cells were grown in HAM‐F12 medium containing 10% fetal bovine serum and 1% penicillin–streptomycin in a humidified atmosphere at 37 °C. Each cell line was used from passage 5 to passage 20. Determination of cell growth was performed using the MTT (3‐(4,5‐dimethylthiazol‐2‐yl)‐2,5‐diphenyltetrazolium bromide) assay. 20 000 cells per well were seeded into 96‐well plates in a final volume of 100 µL, and the various compound concentrations were added in a volume of 50 µL. Stock solution was prepared just before its use. After 48 h, MTT (10 µL, 0.5 mg mL^−1^) was added to each well. The supernatant was removed after 3–4 h of incubation at 37 °C. The formazan crystals were allowed to solubilize using 100 µL of DMSO:EtOH (1:1), and absorbance values (*l*_570_) were determined on a Victor 3 (PerkinElmer Life Sciences) microplate reader. The absorbance of the untreated cells was defined as 100% cell viability; the viability of cells incubated with drugs was measured in each experimental condition and expressed as a percentage (%) of viable cells in the defined condition versus the vitality of the untreated cells. EC_50_ values were determined by graphical analysis using GraphPad Prism 8.4.3 (GraphPad Software, La Jolla, CA).

### Statistical Analysis

Bioactivity data are presented as mean ± standard error of the mean (SEM) of a number of *n* independent experiments. Outliers were determined using Grubb's test. Neither were the sample sizes pre‐determined by statistical methods, samples blinded, nor data confirmed as normally distributed. Different groups were compared by repeated measures one‐way or two‐way ANOVA followed by Tukey HSD post hoc test or by two‐tailed paired student *t*‐test using a two‐sided *α* level of 0.05. *P* values < 0.05 were considered statistically significant. Statistics were calculated and IC_50_ values determined by graphical analysis using GraphPad Prism 8.4.3 (GraphPad Software, La Jolla, CA).

## Conflict of Interest

G.S. declares a potential financial conflict of interest as a co‐founder of inSili.com LLC, Zurich, and in his role as a consultant to the pharmaceutical industry.

## Author Contributions

L.F. and V.B. generated, synthesized, and characterized the computational designs. G.C., M.I., and Y.‐H.K. designed and performed the crystallographic studies. M.G.P. and A.S. performed and analyzed enzymatic and cytotoxicity assays. D.M. performed the nuclear receptor activation assays. A.K., O.W., M.M., and M.G.P. designed and performed the experiments on cyclooxygenase inhibition. K.N. analyzed the metabololipidomics data. M.M. and M.G.P. carried out the cytotoxicity assays. R.K.H. performed activity assays. A.S. supervised the work of M.I., M.M., and M.G.P. G.S. designed the study and wrote the manuscript. All authors analyzed and interpreted the results and contributed to the manuscript.

## Supporting information

Supporting Information

## Data Availability

PDB‐ID: 7JXT. Protein Data Bank: www.pdb.org.
