# Supplementary material for: Learning from Nature: From a Marine Natural Product to Synthetic Cyclooxygenase‐1 Inhibitors by Automated De Novo Design
Source: Adv Sci (Weinh). 2021 Jun 27;8(16):2100832. doi: 10.1002/advs.202100832 (PMC8373093; doi:10.1002/advs.202100832)
Supplement: Supplementary file 1 — Supporting Information [file ADVS-8-2100832-s001.pdf]

© 2021 Wiley-VCH GmbH

ADVANCED SCIENCE
Open Access

Supporting Information

for *Adv. Sci.,* DOI: 10.1002/advs.202100832

Learning from nature: From a marine natural product to synthetic cyclooxygenase-1 inhibitors by automated de novo design

Lukas Friedrich, Gino Cingolani, Ying-Hui Ko, Mariaclara Iaselli, Morena Miciaccia, Maria Grazia Perrone, Konstantin Neukirch, Veronika Bobinger, Daniel Merk, Robert Klaus Hofstetter, Oliver Werz, Andreas Koeberle, Antonio Scilimati & Gisbert Schneider

Supporting Information

Learning from nature: From a marine natural product to synthetic cyclooxygenase-1 inhibitors by automated de novo design

Lukas Friedrich, Gino Cingolani, Ying-Hui Ko, Mariaclara Iaselli, Morena Miciaccia, Maria Grazia Perrone, Konstantin Neukirch, Veronika Bobinger, Daniel Merk, Robert Klaus Hofstetter, Oliver Werz, Andreas Koeberle, Antonio Scilimati & Gisbert Schneider

Contents

Molecular scaffolds and frameworks S2
Top-ranking *de novo* designed molecules S3
Best-scoring 2,4,5-triphenyl imidazole designs S6
Predicted targets and target families S7
In vitro assay results S9
X-ray data collection and refinement statistics S15
^{1}H and ^{13}C NMR spectra of synthesized compounds S16
Infrared spectra of synthesized compounds **2** and **2a** S22
Ultraviolet-visible spectra of synthesized compounds **2** and **2a** S23

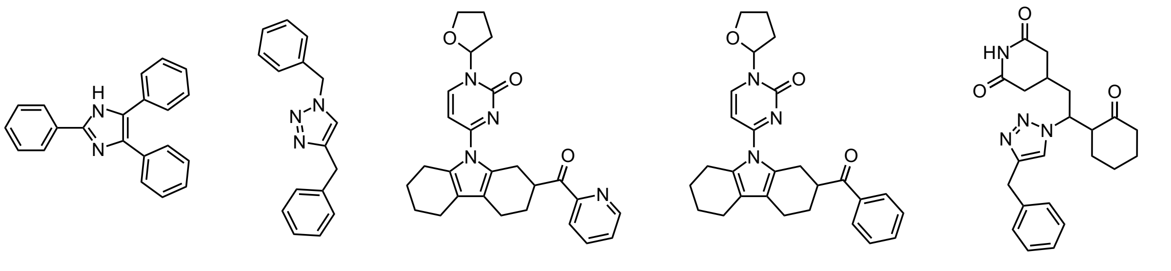

N=122 18 17 13 9

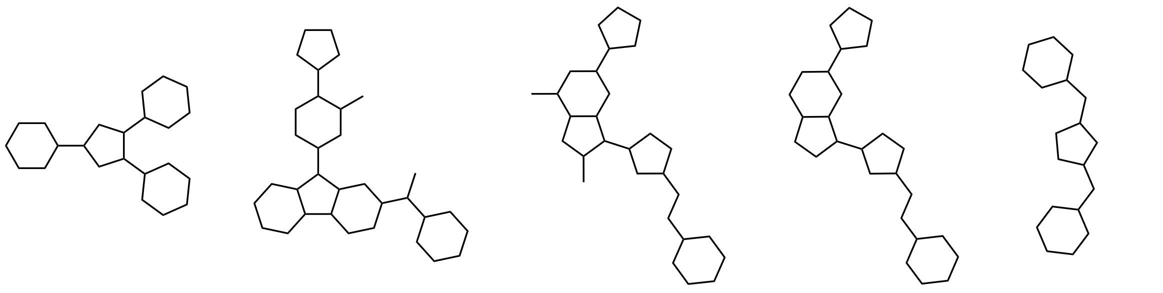

122 45 20 20 18

Figure S1. Five most frequent scaffolds (top) and corresponding molecular graph frameworks (bottom) of the *de novo* generated molecules (802 designs).

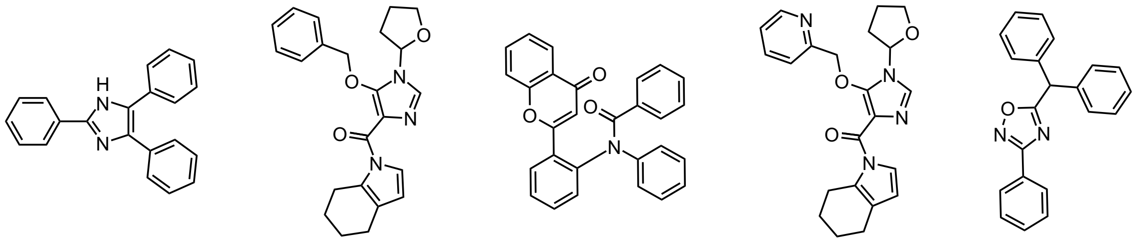

N= 34 9 4 3 3

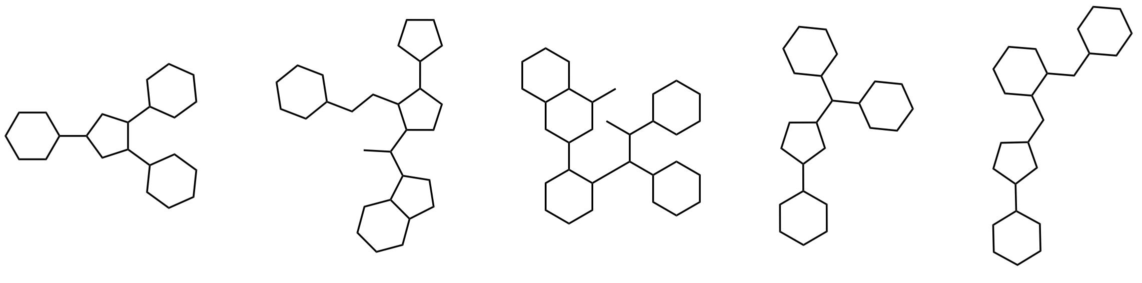

N= 34 12 4 3 3

Figure S2. Five most frequent scaffolds and frameworks of the 100 top-ranked designs according to their CATS distance to marinopyrrole A.

Table S1. 100 top-ranking *de novo* designed molecules according to their CATS distance to marinopyrrole A. The selected compounds for synthesis are on ranks 7 and 56 of this list, respectively.

Rank	SMILES	CATS distance
Rank 1	Oc(cccc1)c1C(N(c1c(Cc([nH]c2c3)nc2cc(Cl)c3Cl)cccc1)c(c(Cl)ccc1)c1Cl)=O	1.241
Rank 2	Cc(c(Cl)c1N(C(c(cccc2)c2O)=O)c(cccc2)c2C(Oc(cc2C)c3cc2Cl)=CC3=O)ccc 1Cl	1.293
Rank 3	Oc(cccc1)c1C(CC(C(Oc1c2O)=O)=C(c(cccc3)c3Cl)c1cc1c2oc(C(c(cc2)cc(Cl) c2Cl)=O)c1)=O	1.388
Rank 4	Cc1ccc(C)n1-c(c(C)c1)cc(-c2noc([C@@H](O)Cl)n2)c1NC(c1ccccc1)=O	1.421
Rank 5	Cc(cccc1)c1C(c(c(O1)c2O)cc3c2oc(C(c(cc2)cc(Cl)c2Cl)=O)c3)=C(CC(c(cccc 2)c2O)=O)C1=O	1.433
Rank 6	Cc(c(Cl)c1N(C(c(cccc2)c2O)=O)c(cccc2)c2-c2nc(cc(cc3)C(c4ccccc4)=O)c3[nH]2)ccc1Cl	1.443
**Rank 7**	**C=CCc1cccc(-c2nc(-c(cccc3)c3Cl)c(-c(cccc3)c3Cl)[nH]2)c1OC(CO)=O**	**1.447**
Rank 8	OCC#CC(c(cc1)cc(-c2nc(-c(cccc3)c3Cl)c(-c(cccc3)c3Cl)[nH]2)c1O)=O	1.465
Rank 9	Cc1ccc(C)n1-c(c(C)c1)cc(-c2noc([C@@H](O)Br)n2)c1NC(c1ccccc1)=O	1.471
Rank 10	Cc1c(C)n(C(c(ncn2C(C(C3O)O)OC3C#N)c2OCc2cc(O)ccc2)=O)c2c1CCCC 2	1.481
Rank 11	Cc(c(C)c1)cc2c1nc(-c(cc1C(c3c4c(O)ccc3)=O)cc(O)c1C4=O)n2-c1cccc(Cl)c1Cl	1.485
Rank 12	Cc(c(Cl)c1Nc(cccc2)c2-c2nc(-c(cc3OC)cc(Br)c3O)no2)ccc1Cl	1.495
Rank 13	Cc1c(C)n(C(c(ncn2C(C3O)OC(CO)C3O)c2OCc2cc(O)ccc2)=O)c2c1CCCC2	1.498
Rank 14	Cc1c(C)n(C(c(ncn2C(C(C3O)O)OC3C#N)c2OCc(cccc2)c2O)=O)c2c1CCCC 2	1.511
Rank 15	Cc1c(C)n(C(c(ncn2C(C(C3O)O)OC3C#N)c2OCc2ncccc2O)=O)c2c1CCCC2	1.516
Rank 16	CC(c(ccc(-c1nc(-c(cccc2)c2Cl)c(-c(cccc2)c2Cl)[nH]1)c1O)c1O1)=CC1=O	1.516
Rank 17	Cc1c(C)n(C(c(ncn2C(C3O)OC(CO)C3O)c2OCc(cccc2)c2O)=O)c2c1CCCC2	1.518
Rank 18	O=C(CCl)Oc(c(F)cc(F)c1)c1-c1nc(-c(cccc2)c2Cl)c(-c(cccc2)c2Cl)[nH]1	1.523
Rank 19	Cc1c(C)n(C(c(ncn2C(C3O)OC(CO)C3O)c2OCc2ncccc2O)=O)c2c1CCCC2	1.523
Rank 20	Cc1c(C)n(C(c(ncn2C(C3O)OC(CO)C3O)c2OCc(cc2)ccc2O)=O)c2c1CCCC2	1.532
Rank 21	Cc1c(C)n(C(c(ncn2C(C3Cl)OC(CCl)C3Cl)c2OCc(cccc2)c2O)=O)c2c1CCCC 2	1.540
Rank 22	Cc(cc1)c(C)c(Oc2c([C@H](c3nc(-c(c(O)ccc4)c4O)no3)Cl)cccc22)c1C2=O	1.544
Rank 23	Cc1c(C)n(C(c(ncn2C(C3Cl)OC(CCl)C3Cl)c2OCc2ncccc2O)=O)c2c1CCCC2	1.545
Rank 24	Cc1c(C)n(C(c(ncn2C(C(C3O)O)OC3C#N)c2OCc(cc2)ccc2O)=O)c2c1CCCC 2	1.546
Rank 25	CC(n(c1c2)c(-c(cccc3)c3N(C(c(cccc3)c3O)=O)c3c(C)c(C)ccc3)nc1cc(Cl)c2Cl)=O	1.548
Rank 26	Cc1c(C)n(C(c(ncn2C(C3Cl)OC(CCl)C3Cl)c2OCc2cc(O)ccc2)=O)c2c1CCCC 2	1.551
Rank 27	CC(n(c1c2)c(-c(cccc3)c3N(C(c(cccc3)c3O)=O)c3c(C)c(Cl)ccc3)nc1cc(Cl)c2Cl)=O	1.552
Rank 28	Oc(cccc1)c1C(N([C@@H]1c(cc(cc2)N(C=Nc3c4cccc3)C4=O)c2-c2c1cccc2)c(c(Cl)c(cc1Cl)Cl)c1Cl)=O	1.568
Rank 29	Oc(c(C=O)cc(-c1noc([C@@H](c(cccc2)c2Nc(c(Cl)ccc2)c2Cl)Cl)n1)c1)c1F	1.578
Rank 30	O=C(Cl)Oc(c(-c1nc(-c(cccc2)c2Cl)c(-c(cccc2)c2Cl)[nH]1)cc(F)c1)c1F	1.581
Rank 31	O=C(CBr)Oc(c(-c1nc(-c(cccc2)c2Cl)c(-c(cccc2)c2Cl)[nH]1)cc(F)c1)c1F	1.581
Rank 32	Cc(cc(c1c2)OC(c(cccc3)c3N(C(c(cccc3)c3O)=O)c3c(C)c(Cl)ccc3)=CC1=O)c 2Cl	1.589

Rank 33	OCC#CC(c(cc1)cc(O)c1-c1nc(-c(cccc2)c2Cl)c(-c(cccc2)c2Cl)[nH]1)=O	1.589
Rank 34	Cc1cccc(N(C(c2ccc[nH]2)=O)c(cccc2)c2C(Oc2c3ccc(O)c2C)=CC3=O)c1C	1.597
Rank 35	Oc(c(C=O)cc(F)c1)c1-c1noc(C(c(cccc2)c2Cl)(c(cccc2)c2Cl)Cl)n1	1.606
Rank 36	Cc(c(OC(c(cccc1)c1N(C(c1ccc[nH]1)=O)c1cc(Cl)cc(Cl)c1)=C1)c(cc2)C1=O)c2O	1.609
Rank 37	OCC(Oc(c(-c1nc(-c(cccc2)c2Cl)c(-c(cccc2)c2Cl)[nH]1)cc(Cl)c1)c1Br)=O	1.616
Rank 38	Cc1c(C)n(C(c(ncn2C(C3Cl)OC(CCl)C3Cl)c2OCc(cc2)ccc2O)=O)c2c1CCCC2	1.621
Rank 39	OCC(Oc(c(-c1nc(-c(cccc2)c2Cl)c(-c(cccc2)c2Cl)[nH]1)cc(Cl)c1)c1Cl)=O	1.628
Rank 40	OCc(c1c2CCCC1)c(CO)n2-c(cccc1)c1S(Nc1cccc2cccnc12)(=O)=O	1.630
Rank 41	Oc(c(C=O)cc(F)c1)c1-c1noc(C(c(cccc2)c2Cl)(c(cccc2)c2Cl)Br)n1	1.632
Rank 42	Cc1ccc(C)n1CC(C(C1O)O)OC1n1c2ncnc(-n3c(C)ccc3C)c2nc1	1.637
Rank 43	Cc1cccc(N(C(c(cccc2)c2O)=O)c(cccc2)c2C(Oc(c2c3)cc(C)c3Cl)=CC2=O)c1C	1.644
Rank 44	Cc(nc1C(c(cccc2)c2Nc(c2c3cc(-c(c(Cl)ccc4F)c4Cl)[nH]2)ccc3Cl)=O)ccc1O	1.645
Rank 45	Cc1ccc(C)n1-c(cc(cc1)Cl)c1N(C(c(cccc1)c1O)=O)c(cccc1)c1C(Oc(c1c2)cc(C)c2Cl)=CC1=O	1.650
Rank 46	CCOc1cccc(-c2nc(-c(cccc3)c3Cl)c(-c(cccc3)c3Cl)[nH]2)c1O	1.656
Rank 47	CC(Oc(c(-c1nc(-c(cccc2)c2Cl)c(-c(cccc2)c2Cl)[nH]1)cc(F)c1)c1Cl)=O	1.659
Rank 48	CC(Oc(cccc1-c2nc(-c(cccc3)c3Cl)c(-c(cccc3)c3Cl)[nH]2)c1Cl)=O	1.663
Rank 49	Cc(cc(c(C(c(cccc1)c1Cl)=O)c1)OCc(cc2C(c3c4c(O)ccc3)=O)cc(O)c2C4=O)c1Cl	1.666
Rank 50	Cc(c(Cl)c1N(C(c(cccc2)c2N)=O)c(cccc2)c2C(Oc(cc2C)c3cc2Cl)=CC3=O)ccc1Cl	1.669
Rank 51	CC(Oc(c(C)cc(F)c1)c1-c1nc(-c(cccc2)c2Cl)c(-c(cccc2)c2Cl)[nH]1)=O	1.670
Rank 52	OCC(Oc(c(-c1nc(-c(cccc2)c2Cl)c(-c(cccc2)c2Cl)[nH]1)cc(Br)c1)c1Br)=O	1.672
Rank 53	CC(Oc(ccc(Cl)c1)c1-c1nc(-c(cccc2)c2Cl)c(-c(cccc2)c2Cl)[nH]1)=O	1.680
Rank 54	CC(Oc(c(-c1nc(-c(cccc2)c2Cl)c(-c(cccc2)c2Cl)[nH]1)ccc1)c1Cl)=O	1.681
Rank 55	CC(C)(C)Oc(cc(cc1)-c2nc(-c(cccc3)c3Cl)c(-c(cccc3)c3Cl)[nH]2)c1O	1.683
**Rank 56**	**CCOc(cc(cc1)-c2nc(-c(cccc3)c3Cl)c(-c(cccc3)c3Cl)[nH]2)c1O**	**1.699**
Rank 57	Cc1ccc(C)n1C(NC1=O)=Nc2c1ncn2C(C1Cl)OC(COc(cc2)ncc2I)C1Cl	1.706
Rank 58	Cc(cc1)c(C)c(Oc2c([C@H](c3nc(-c(c(O)ccc4)c4O)no3)Br)cccc22)c1C2=O	1.707
Rank 59	N#Cc(cc1F)cc(-c2nc(-c(cccc3)c3Cl)c(-c(cccc3)c3Cl)[nH]2)c1O	1.708
Rank 60	Cc1cccc(-n2c(-c(cc3C(c4cccc(O)c44)=O)cc(O)c3C4=O)nc3c2cc(C)c(C)c3)c1C	1.709
Rank 61	Cc1ccc(C)n1-c1c(C(N(C=Nc2c3cccc2)C3=O)=O)ncn1C(C1O)OC(CO)C1O	1.716
Rank 62	Oc(cc1)cc2c1[nH]c1c2CC(C(Cl)=O)NC1c1cccc(Oc(cc2)cc(Cl)c2Cl)c1	1.717
Rank 63	CC(c1cc(-c2noc(C(c(cccc3)c3Cl)(c(cccc3)c3Cl)O)n2)c(C)cc1O)=O	1.721
Rank 64	Oc(c(C(N(CC1)[C@@H](c2c(-c3ccccc3)[nH]c3c2cccc3)c2c1c(Cl)cc(Cl)c2)=O)c1)cc(O)c1Br	1.723
Rank 65	CC(Oc(c(F)cc(-c1nc(-c(cccc2)c2Cl)c(-c(cccc2)c2Cl)[nH]1)c1)c1Cl)=O	1.723
Rank 66	Cc1ccc(C)n1C(NC1=O)=Nc2c1ncn2C(C1Cl)OC(COc2cccnc2I)C1Cl	1.726
Rank 67	CC(C)(C)c(cc1)ccc1-c1nc(-c(cc2C(c3cccc(O)c33)=O)cc(O)c2C3=O)n[nH]1	1.729
Rank 68	Nc(cccc1)c1C(N(c1c(Cc([nH]c2c3)nc2cc(Cl)c3Cl)cccc1)c(c(Cl)ccc1)c1Cl)=O	1.730
Rank 69	Oc(c(C=O)cc(-c1noc([C@@H](c(cccc2)c2Nc(c(Cl)ccc2)c2Cl)Br)n1)c1)c1F	1.732
Rank 70	COc(cc1)ccc1-c(c1c2CCCC1)c(-c(cc1)ccc1OC)n2C(C(Cl)=CN1C(C2O)OC(CO)C2O)=NC1=O	1.734

Rank 71	Oc(ccc(-c1cc(c(CCCl)c(cc2)N(C(c3c4cccc3)=O)C4=O)c2[nH]1)c1)c1O	1.734
Rank 72	CC(Oc(ccc(-c1nc(-c(cccc2)c2Cl)c(-c(cccc2)c2Cl)[nH]1)c1)c1Cl)=O	1.735
Rank 73	COc1cc(-c2noc(Cc(cccc3)c3Nc(c(Cl)ccc3)c3Cl)n2)cc(Br)c1O	1.736
Rank 74	Cc(c(C)c1)cc(C)c1-c1cn(Cc(cc2C(c3cccc(O)c33)=O)cc(O)c2C3=O)nn1	1.749
Rank 75	Cc(cc1)c(C)c(Oc2c([C@H](c3nc(cc(cc4)C(c5ccccc5)=O)c4[nH]3)Cl)cccc22)c 1C2=O	1.753
Rank 76	Cc1ccc(C)n1-c1c(C(N(C=Nc2c3cccc2)C3=O)=O)ncn1C(C(C1O)O)OC1C#N	1.756
Rank 77	CC(c1cc(-c2noc(-c(cccc3)c3Nc3cccc(Cl)c3C)n2)c(C)cc1O)=O	1.756
Rank 78	Clc(cccc1)c1-c1c(-c(cccc2)c2Cl)nc(-c(cc2)cc(Cl)c2Cl)[nH]1	1.760
Rank 79	Cc1c(C)c(- c(c(C)c2)cc3c2OC(c(cc2C(c4cccc(O)c44)=O)cc(O)c2C4=O)=CC3=O)c(C)c(C)c1	1.761
Rank 80	CC(Oc(c(C)ccc1)c1-c1nc(-c(cccc2)c2Cl)c(-c(cccc2)c2Cl)[nH]1)=O	1.764
Rank 81	CC(Oc(c(-c1nc(-c(cccc2)c2Cl)c(-c(cccc2)c2Cl)[nH]1)ccc1)c1Br)=O	1.764
Rank 82	O=C(Cl)Oc(c(-c1nc(-c2ccccc2)c(-c(cc2)ccc2Cl)[nH]1)cc(Br)c1)c1F	1.764
Rank 83	OCC(Nc(c(-c1nc(-c(cccc2)c2Cl)c(-c(cccc2)c2Cl)[nH]1)cc(Br)c1)c1Br)=O	1.765
Rank 84	Cc1ccc(c(Cl)cc(Cl)c2OCc(cc3C(c4c5c(O)ccc4)=O)cc(O)c3C5=O)c2n1	1.767
Rank 85	COc(cc1)ccc1-c(c1c2CCCC1)c(- c(cc1)ccc1OC)n2C(C(Cl)=CN1C(C(C2O)O)OC2C#N)=NC1=O	1.767
Rank 86	Oc(cc1)cc(-c2nc(-c(cccc3)c3Cl)c(-c(cccc3)c3Cl)[nH]2)c1O	1.767
Rank 87	Oc1c(C(c(cccc2)c2O)N(C(c2ccc[nH]2)=O)c(c(Cl)c(cc2Cl)Cl)c2Cl)cccc1	1.773
Rank 88	CC(C)OC(c(cc(cc1)-c2nc(-c(cccc3)c3Cl)c(-c(cccc3)c3Cl)[nH]2)c1O)=O	1.775
Rank 89	CC(Oc(ccc(C)c1)c1-c1nc(-c(cccc2)c2Cl)c(-c(cccc2)c2Cl)[nH]1)=O	1.777
Rank 90	CC(Oc(ccc(I)c1)c1-c1nc(-c(cccc2)c2Cl)c(-c(cccc2)c2Cl)[nH]1)=O	1.777
Rank 91	CC(Oc(ccc(Br)c1)c1-c1nc(-c(cccc2)c2Cl)c(-c(cccc2)c2Cl)[nH]1)=O	1.777
Rank 92	N#Cc1cc(-c2nc(-c(cccc3)c3Cl)c(-c(cccc3)c3Cl)[nH]2)cc(F)c1O	1.778
Rank 93	Oc(cc1)cc2c1[nH]c1c2CC(C(Cl)=O)NC1c1cccc(Oc2cc(Cl)cc(Cl)c2)c1	1.784
Rank 94	C[C@@H](C#CC(c(cc(cc1)-c2nc(-c3ccccc3)c(- c(cc3)ccc3Cl)[nH]2)c1O)=O)O	1.786
Rank 95	CC(C)C(c1cc(-c2nc(-c3ccccc3)c(-c(cc3)ccc3Cl)[nH]2)cc(OC)c1O)=O	1.789
Rank 96	COC(/C=C/c(cc1)ccc1-c1nc(-c(cccc2)c2Cl)c(-c(cccc2)c2Cl)[nH]1)=O	1.792
Rank 97	Cc1ccc(C)n1CC(C(C1Cl)Cl)OC1n1c2ncnc(-n3c(C)ccc3C)c2nc1	1.794
Rank 98	CC(C)(C)c(cc1)cc2c1oc(-c(cc1C(c3cccc(O)c33)=O)cc(O)c1C3=O)n2	1.794
Rank 99	Cc(c(C(c(cc(c(Cl)n1)F)c1Cl)=C1)c(c(C(c(cccc2)c2Cl)=O)c2)OC1=O)c2Cl	1.796
Rank 100	CC(n(c1c2)c(-c(cccc3)c3C(c(cc3)ccc3Cl)=O)nc1cc(Cl)c2Cl)=O	1.798

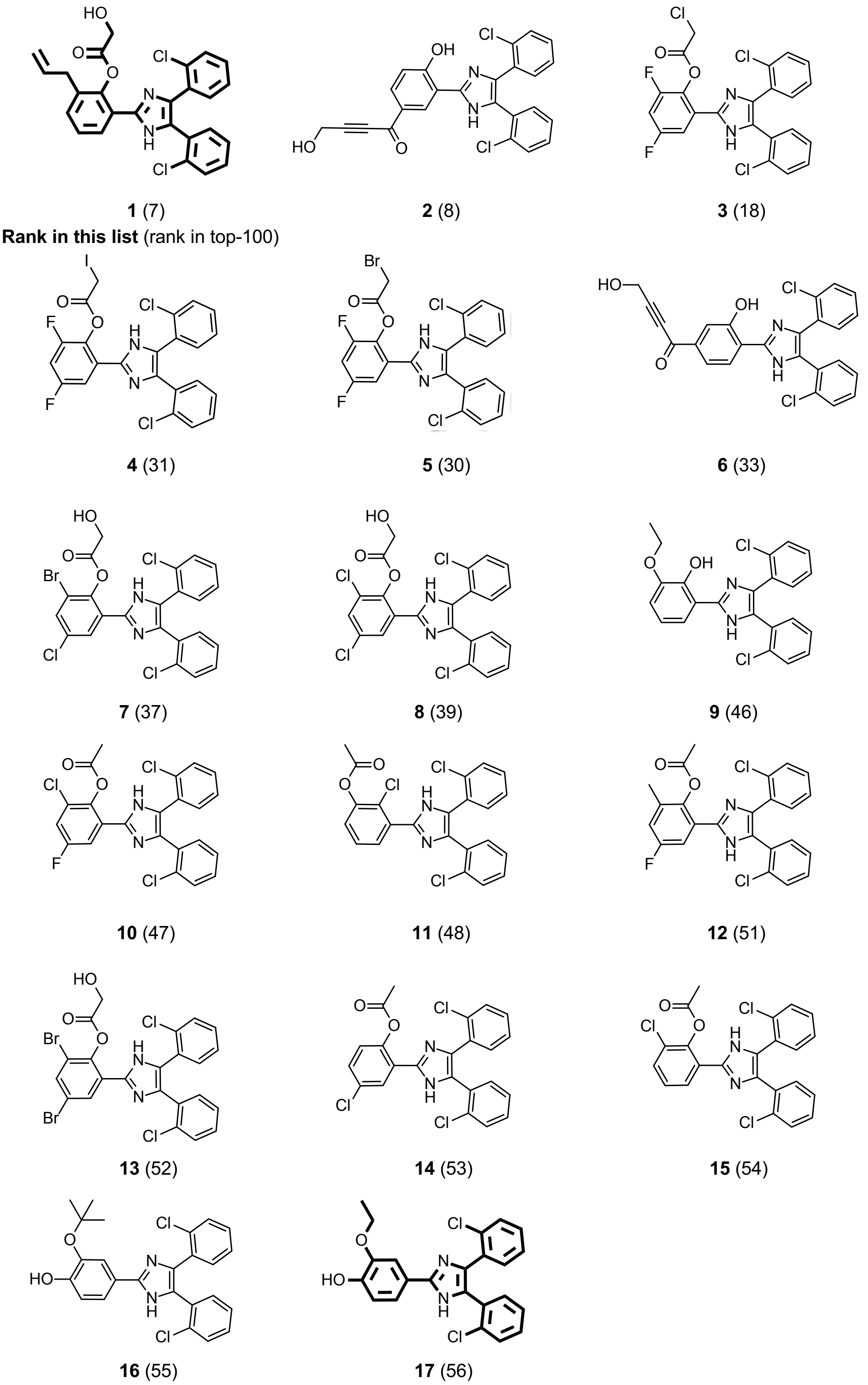

Figure S3. The 17 best-scoring designs among the 100 top-ranked *de novo* designs with a 2,4,5-triphenyl imidazole scaffold. Numbers indicate ranks. The molecules ranked 1st and 17th (highlighted) on this list were synthesized and tested. Note: These rank numbers **1–17** differ from the compound identifiers (Arabic numbers in the main article) of Marinopyrrole A and the synthesized designed molecules.

Table S2. Predicted targets and target families of the compounds according to SPiDER software. Predictions with *p* ≤0.5 are listed.

ACAT, Acyl-CoenzymeA:Cholesterol O-Acyltransferase; AHR, Aryl HydrocarbonR; CAI, Capsid Assembly Inhibitor; CB, CannabinoidR; CFTR, Cystic Fibrosis Transmembrane Conductance Regulator; CRF, Corticotropin-Releasing Factor Receptor; DHODA, Dihydroorotate Dehydrogenase; FTase, Farnesyltransferase; GPBAR1, G-Protein Coupled Bile Acid Receptor 1; HCA, Hydroxycarboxylic Acid Receptor; mGluR, Metabotropic Glutamate Receptor; nAChR, Nicotinic Acetylcholine Receptor; GABA, Gamma-Aminobutyric Acid Receptor; PDE, Phosphodiesterase; PPAR, Peroxisome Proliferator-Activated Receptor; TNFalpha, Tumor Necrosis Factor-Alpha; TRP, Transient Receptor Potential Ion Channel; TS, Thymidylate Synthase

**Compound 1**	*p*	**Compound 2**	*p*	**Compound 2a**	*p*	**Compound 2b**	*p*	**Compound 3**	*p*	**Compound 4**	*p*
ProstanoidR	0.026	InterleukinR	0.006	InterleukinR	0.006	DNA Polym.	0.006	CRF	0.002	CRF	0.014
CB	0.031	ProstanoidR	0.006	Cyclooxygenase	0.007	InterleukinR	0.007	EstrogenR	0.003	DNA Polym.	0.017
PPAR	0.045	DNA Polym.	0.007	ProstanoidR	0.007	ProstanoidR	0.009	MineralcorticoidR	0.004	TNFalpha	0.019
CRF	0.047	CAI	0.009	DNA Polym.	0.009	Cyclooxygenase	0.01	Leukotriene	0.004	PPAR	0.02
Ser/Thr kinase	0.048	Cyclooxygenase	0.01	CAI	0.009	CAI	0.011	TS	0.004	ProstanoidR	0.022
RNA Polym.	0.048	Endopeptidase	0.014	PPAR	0.015	CRF	0.011	nAChR	0.006	Ser/Thr kinase	0.024
Cyclooxygenase	0.05	TNFalpha	0.015	GlucagonR	0.015	AdenosineR	0.013	DHODH	0.006	CAI	0.024
GlucagonR	0.05	GlucocorticoidR	0.015	GlucocorticoidR	0.016	GlucagonR	0.015	NGABA	0.007	CB	0.024
		PPAR	0.016	CRF	0.016	CB	0.016	Neuropeptide Y R	0.007	GlucagonR	0.025
		CB	0.016	CB	0.016	PPAR	0.016	PPAR	0.01	Endopeptidase	0.025
		GlucagonR	0.017	TNFalpha	0.016	Endopeptidase	0.016	Tyrosine Kinase	0.01	Cyclooxygenase	0.026
		AdenosineR	0.017	AdenosineR	0.017	K+ channel	0.017	Endopeptidase	0.01	AdenosineR	0.026
		Ser/Thr kinase	0.017	AnaphylatoxinR	0.019	Ser/Thr kinase	0.018	DNA Polym.	0.01	GlucocorticoidR	0.026
		CRF	0.018	Ser/Thr kinase	0.019	TNFalpha	0.019	Cyclooxygenase	0.012	Endopeptidase	0.027
		Mitotic Kinesin	0.02	Endopeptidase	0.02	AnaphylatoxinR	0.022	mGluR	0.013	K+ channel	0.029
		Endopeptidase	0.021	K+ channel	0.02	Endopeptidase	0.023	Ser/Thr kinase	0.013	Phospholipase	0.03
		AnaphylatoxinR	0.021	Endopeptidase	0.023	PDE	0.025	AdenosineR	0.014	Tyrosine Kinase	0.033
		K+ channel	0.022	Mitotic Kinesin	0.025	GlucocorticoidR	0.026	TRP	0.014	AnaphylatoxinR	0.035
		PDE	0.027	PDE	0.027	Mitotic Kinesin	0.028	Cyp P450	0.014	Mitotic Kinesin	0.037
		Tyrosine Kinase	0.031	DNA Topoisom.	0.03	Tyrosine Kinase	0.029	ProstanoidR	0.014	PDE	0.038
		DNA Topoisom.	0.031	ACAT	0.03	ACAT	0.03	Endopeptidase	0.014	ACAT	0.042
		VasopressinR	0.032	Tyrosine Kinase	0.03	DNA Topoisom.	0.031	Smoothened	0.015	DNA Topoisom.	0.045
		ACAT	0.035	VasopressinR	0.036	Phospholipase	0.038	HCA	0.015	VasopressinR	0.05
		Phospholipase	0.036	Phospholipase	0.041	VasopressinR	0.042	CAI	0.015		
		Liver X R	0.042	Liver X R	0.042	Liver X R	0.05	VasopressinR	0.018		

Na+ channel	0.018
DNA Topoisom.	0.021
PDE	0.022
GlucagonR	0.022
CholecystokininR	0.022
Liver X R	0.022
Thymidine Kinase	0.026
FTase	0.027
Mitotic Kinesin	0.028
K+ channel	0.03
CB	0.031
TachykininR	0.031
CFTR	0.034
EndothelinR	0.035
Integrins	0.037
Ligase	0.04
GPBAR1	0.041
Phospholipase	0.05

Table S3. Preliminary screening results provided by Eurofins (Cerep, France). Values are reported as the mean effect (agonist assays), inhibition (antagonist assays and enzymatic assays) or binding competition effects (binding assays) in percent. *n.d.*, not determined.

Target (mode of action)	Mean % effect (n = 2)					
	**1**	**2**	**2a**	**2b**	**3**	**4**
COX-1 (enzymatic)	**95**	*n.d.*	**95**	**78**	**98**	**98**
EP1 (antagonism)	**86**	**73**	3	-4	19	11
EP2 (antagonism)	**104**	15	-30	10	**34**	**35**
EP3 (antagonism)	**84**	**46**	20	13	**41**	13
EP4 (antagonism)	**37**	7	-10	-21	-25	3
CB1 (agonism)	**43**	-4				
CB1 (antagonism)	-7	**86**				
CB2 (agonism)	**31**	-12		*n.d.*		
CB2 (antagonism)	14	**59**				
CRF1(antagonism)	**75**	**57**	7	28	**45**	**46**
CCK2 (antagonism)	1	**64**	11	14	**66**	36
GR (binding competition)	**98**	**85**	27	22	**59**	**86**
OX1 (antagonism)	**116**	**66**	14	8	**57**	6
OX2 (antagonism)	**103**	**63**	15	9	**37**	11

COX, cyclooxygenase; EP, prostaglandin E_2R; CB, cannabinoid receptor; CRF, corticotropin-releasing factor receptor; CCK, cholecystokinin B receptor; GR, glucocorticoid receptor; OX, orexin receptor

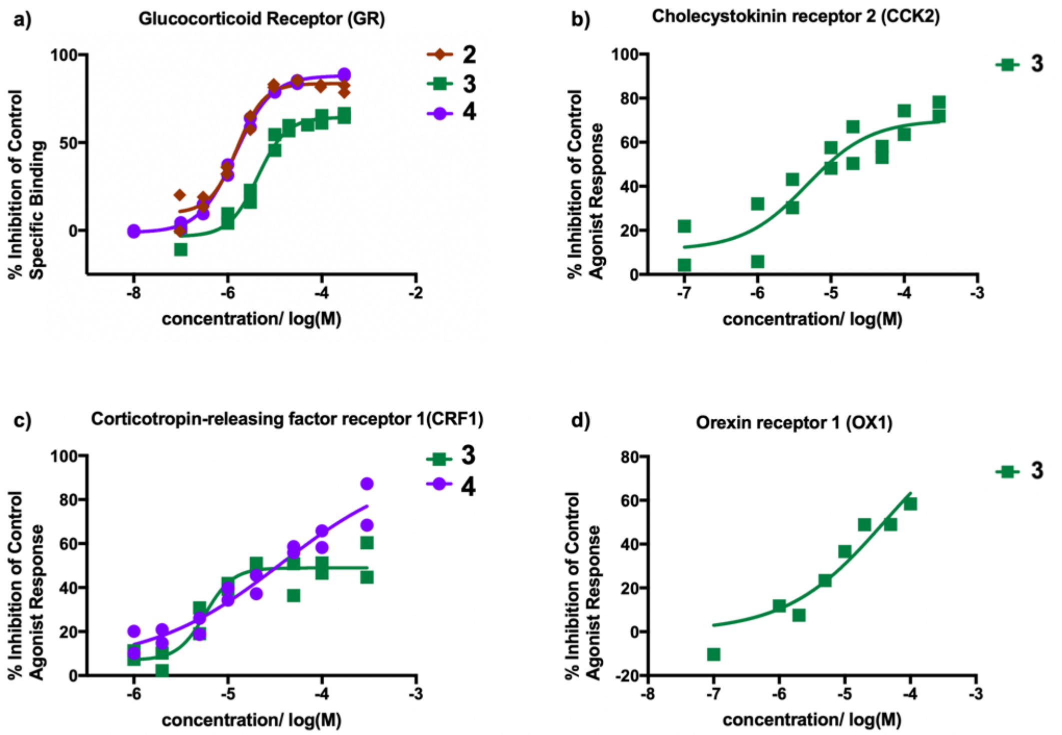

Figure S4. ***In vitro*** **activity of compounds 2, 3, and 4 on GR, CCK2, CRF1, and OX1. a) 2** (IC_{50} = 1.2±1.2 μM, K_B = 0.6 μM), **3** (IC_{50} = 4.3±1.2 μM, K_B = 2.2 μM) and **4** (IC_{50} = 1.4±1.1 μM, K_B = 0.7 μM) bind to the glucocorticoid receptor (GR) in a competition binding assay. **b) 3** (IC_{50} = 8.7±4.6 μM, K_i = 1.1 μM) is an antagonist of the cholecystokinin receptor 2 (CCK2). **c) 3** (IC_{50} = 4.7±1.2 μM, K_i = 1.7μM) and **4** (IC_{50} = 40±2 μM, K_i = 14 μM) have antagonistic effects on the CRF 1 (CRF1). **d)** Compound **3** (IC_{50} = 40±1 μM, K_i = 8.4 μM) also antagonizes orexin receptor 1 (OX1). All assays n = 2 independent experiments.

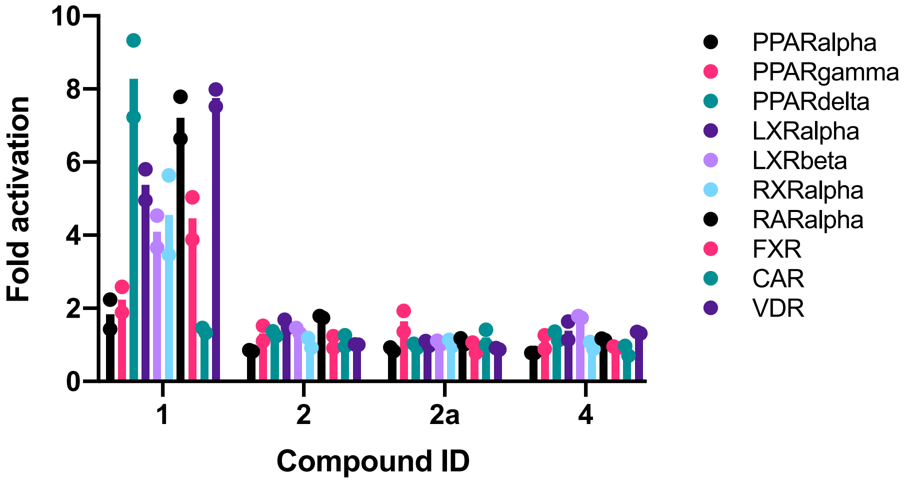

Figure S5. Modulation of nuclear hormone receptors. *In vitro* effect of marinopyrrole A (**1**), the designed compounds **2** and **2a**, and the synthesis intermediate **4** on human nuclear receptors (PPAR, peroxisome proliferator-activated receptor; LXR, liver X receptor; RXR, retinoid X receptor; RAR, retinoic acid receptor; FXR, farnesoid X receptor; CAR, constitutive androstane receptor; VDR, vitamin D receptor). Marinopyrrole A activated several nuclear receptors with low efficiency at a concentration of 10 μM. Mean of n = 2 independent experiments with two technical replicas each.

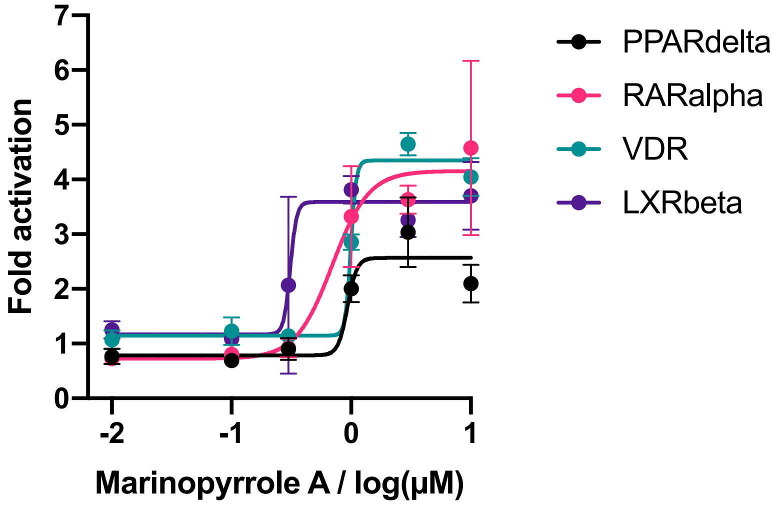

Figure S6. **Effect of marinopyrrole A (compound 1) on selected nuclear hormone receptors**. Marinopyrrole A activates peroxisome proliferator-activated receptor delta (PPARδ, EC_{50} = 0.9±1.2 μM), retinoic acid receptor alpha (RARα, EC_{50} = 0.63±0.11 μM), vitamin D receptor (VDR, EC_{50} = 1.09±0.06 μM), and liver X receptor β (LXRβ, EC_{50} = 0.37±0.40 μM). Mean ± standard error of the mean (s.e.m.) of n = 3 independent experiments. Note: Maximal fold receptor activation of PPARδ differs from Figure S5 because of different assay volumes. The respective EC_{50} value may not be sufficiently confident and should be treated with caution.

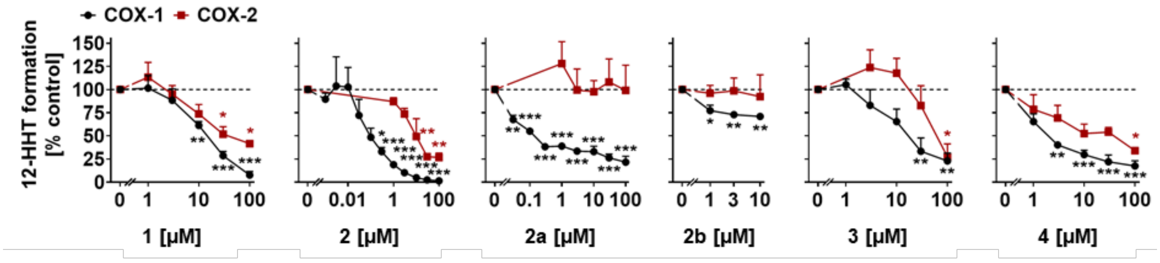

Figure S7. Marinopyrrole A (1) mimetics selectively inhibit COX-1. Isolated ovine COX-1 or human recombinant COX-2 were pre-incubated with **1, 2, 2a, 2b, 3**, or **4**, respectively. The enzymatic reaction was triggered by addition of arachidonic acid, and 12-HHT was analyzed by UV-RP-HPLC. Mean ± standard error of the mean (s.e.m.) of n = 3 independent experiments. *p <0.05, **p <0.01, ***p <0.001 vs vehicle; repeated measures one-way ANOVA and Tukey's *post hoc* test.

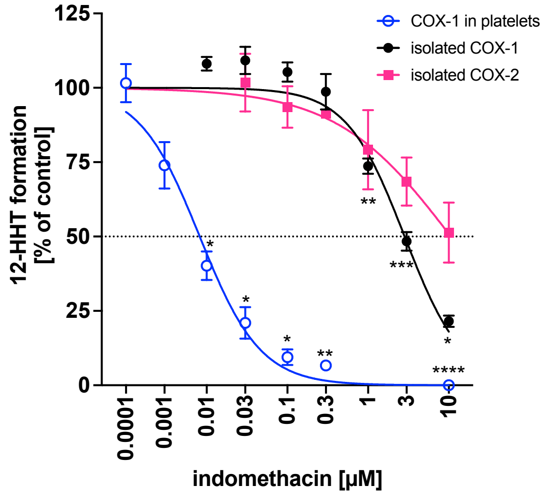

Figure S8. COX inhibition by indomethacin.
See captions of Figure S7 and S10 for details.

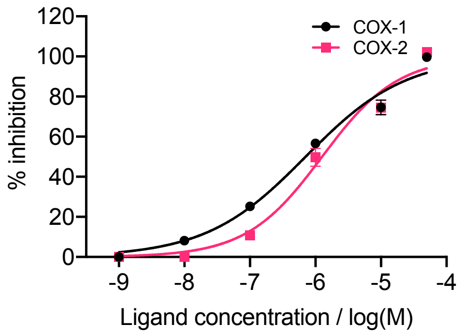

Figure S9. Inhibition of COX endoperoxidase activity by compound 2. The inhibitory effect was determined in a commercial colorimetric test in n = 2 independent experiments. Sigmoidal curve fitting (constraints: top = 100%, bottom = 0%): IC_{50} COX-1 = 0.7±1.2 µM, IC_{50} COX-1 = 1.3±1.2 µM (mean ± standard error of the mean (s.e.m.) of n = 2 independent experiments).

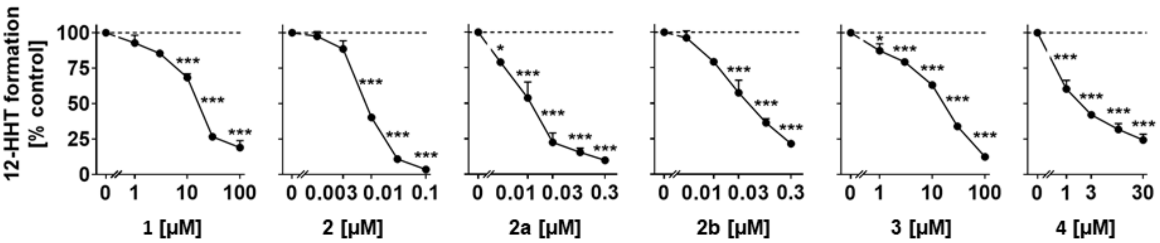

Figure S10. Marinopyrrole A (1) mimetics potently inhibit COX-1 in platelets. Human platelets were pre-incubated with **1**, **2**, **2a, 2b**, **3**, or **4**. COX-1 product formation was triggered by addition of arachidonic acid, and 12-HHT was analyzed by UV-RP-HPLC. Mean ± standard error of the mean (s.e.m.) of n = 3 independent experiments. *p <0.05, **p <0.01, ***p <0.001 vs. vehicle; repeated measures one-way ANOVA + Tukey's *post hoc* test.

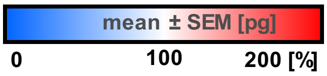

	treatment µM	DMSO 0	2 / µM 0.01	2 / µM 0.1	2 / µM 1	indomethacin 10
COX	PGE2	66.5 ± 2.7	18.9 ± 1.6 ***	6.4 ± 1.0 ***	5.6 ± 1.0 ***	34.8 ± 28.5 ***
	15-ketoPGE2	37.1 ± 5.6	16.0 ± 4.3 **	8.7 ± 2.2 ***	6.2 ± 1.6 ***	20.3 ± 15.3 ***
	PGD2	12.3 ± 0.5	3.3 ± 0.1 ***	1.9 ± 0.2 ***	2.3 ± 0.6 ***	5.8 ± 4.1 ***
	PGF2α	14.8 ± 0.5	6.4 ± 1.6 **	2.4 ± 0.7 ***	2.2 ± 0.2 ***	9.0 ± 5.7 **
	TXB2	4643 ± 298	1108 ± 320 ***	356 ± 102 ***	291 ± 75 ***	2317 ± 2082 ***
	12-HHT	402 ± 43	106 ± 20 ***	32.6 ± 6.6 ***	33.2 ± 2.2 ***	265 ± 240 ***
	11-HETE	154 ± 18	74.2 ± 9.3 *	49.6 ± 3.9 ***	51.5 ± 5.4 ***	109 ± 63
	11-HEPE	5.2 ± 0.7	2.7 ± 0.4 *	2.1 ± 0.1 *	2.4 ± 0.3 *	3.9 ± 1.5
5-LO	LTB4	2532 ± 1804	2911 ± 2281	2624 ± 1937	2499 ± 1910	2083 ± 1374
	t-LTB4	809 ± 561	841 ± 607	821 ± 591	782 ± 590	684 ± 386
	20-OH-LTB4	2394 ± 2195	2686 ± 2491	2359 ± 2159	2546 ± 2324	2366 ± 2130
	5-HETE	2353 ± 1632	2739 ± 2019	2588 ± 1852	2151 ± 1518	1814 ± 1176
	5,15-diHETE	195 ± 106	179 ± 104	177 ± 96	156 ± 85	145 ± 68
	5S,6R-diHETE	380 ± 306	369 ± 303	394 ± 324	246 ± 196	303 ± 253
	5-HEPE	125 ± 97	148 ± 122	129 ± 101	134 ± 106	105 ± 77
12/15-LO	12-HETE	17128 ± 2466	18884 ± 2195	18591 ± 2959	22215 ± 4067	17496 ± 2486
	15-HETE	137 ± 40	106 ± 31	106 ± 40	124 ± 49	116 ± 38
	12-HEPE	564 ± 16	633 ± 72	572 ± 42	676 ± 80	544 ± 18
	15-HEPE	5.6 ± 0.8	5.5 ± 0.8	5.3 ± 1.1	8.7 ± 3.5	5.4 ± 1.0
	14-HDHA	1693 ± 154	1766 ± 256	1700 ± 255	1830 ± 312	1737 ± 213
	17-HDHA	49.7 ± 11.8	53.5 ± 13.1	50.3 ± 12.2	65.1 ± 24.0	51.3 ± 9.4
	14-HDPA	3208 ± 655	3398 ± 848	3312 ± 769	3126 ± 755	3233 ± 776
	LXA4	23.4 ± 15.9	19.1 ± 12.9	20.5 ± 13.6	18.6 ± 14.0	16.4 ± 10.5
others	9-HODE	277 ± 30	152 ± 12	91.6 ± 6.5 ***	133 ± 38 **	196 ± 88
	13-HODE	527 ± 108	505 ± 124	459 ± 142	549 ± 209	534 ± 112
	8-HETE	91.2 ± 2.0	107 ± 10	104 ± 9	119 ± 18	86.9 ± 4.3
	7-HDHA	28.6 ± 17.9	29.1 ± 20.1	28.7 ± 18.4	29.2 ± 16.5	30.1 ± 18.6
	10-HDHA	38.8 ± 2.5	40.2 ± 5.8	38.6 ± 3.6	43.8 ± 6.6	40.5 ± 5.5
	13-HDHA	22.1 ± 0.7	13.6 ± 0.6	9.9 ± 0.9 **	9.7 ± 0.8 **	14.6 ± 3.9
	7-HDPA	28.7 ± 9.5	30.5 ± 10.8	29.6 ± 8.3	30.4 ± 7.7	28.3 ± 9.5
	13-HDPA	398 ± 72	423 ± 90	421 ± 96	403 ± 106	427 ± 103
free PUFA	AA	67596 ± 12215	75244 ± 20248	66713 ± 10824	78579 ± 10313	62361 ± 7082
	EPA	2785 ± 959	3152 ± 1358	2510 ± 925	3234 ± 923	2771 ± 832
	DHA	4401 ± 465	4532 ± 818	4016 ± 425	5022 ± 817	4554 ± 444

Figure S11. Compound 2 preferentially inhibits COX-1 product formation in monocytes. Monocytes were treated with vehicle (DMSO), compound **2**, or indomethacin and activated with A23187. Lipid mediator profiles were analyzed by UPLC-MS/MS. The color scale indicates the percentage changes of lipid mediators vs. vehicle. The values represent mean ± standard error of the mean (s.e.m.) from *n* = 3 independent experiments in pg. **p* <0.05, ***p* <0.01, ****p* <0.001 vs. vehicle; repeated measures two-way ANOVA + Tukey's *post hoc* test of logarithmized data. (di)HETE, (di)hydroxy-eicosatetraenoic acid; HEPE, hydroxy-eicosapentaenoic acid; LT, leukotriene; t-LTB4, trans-LTB4 isomers; 20-OH-LTB4, 20-hydroxy-LTB4; HDHA, hydroxy-docosahexaenoic acid; LX, lipoxin; HODE, hydroxy-octadecadienoic acid; HDPA, hydroxy-docosapentaenoic acid; AA, arachidonic acid; EPA, eicosapentaenoic acid; DHA, docosahexaenoic acid.

mean ± SEM [pg]
0 100 200 [%]

	treatment	DMSO	2 / µM			indomethacin	celecoxib
	µM	0	0.01	0.1	1	10	5
COX	PGE2	898 ± 317	787 ± 332	302 ± 127 ***	118 ± 44 ***	84.7 ± 22.1 ***	122 ± 49 ***
	15-ketoPGE2	150 ± 38	72.1 ± 22.9 **	43.6 ± 11.3 ***	35.3 ± 11.0 ***	35.5 ± 4.8 ***	45.2 ± 9.5 ***
	PGD2	25.5 ± 8.1	19.9 ± 7.1	10.2 ± 3.4 ***	5.1 ± 1.4 ***	5.7 ± 1.0 ***	5.4 ± 1.3 ***
	PGF2α	48.9 ± 21.1	42.3 ± 15.9	18.7 ± 8.0 ***	10.8 ± 3.6 ***	8.9 ± 2.5 ***	11.2 ± 3.8 ***
	TXB2	567 ± 168	140 ± 48 ***	66.8 ± 20.8 ***	35.6 ± 8.7 ***	33.6 ± 7.4 ***	72.0 ± 31.1 ***
	12-HHT	90.3 ± 28.5	29.8 ± 10.2 ***	16.3 ± 5.8 ***	9.7 ± 2.8 ***	10.2 ± 2.2 ***	13.3 ± 4.7 ***
	11-HETE	178 ± 61	164 ± 68	91.7 ± 36.7 *	51.9 ± 19.7 ***	37.2 ± 9.6 ***	47.3 ± 16.1 ***
	11-HEPE	8.3 ± 2.7	5.6 ± 1.9	2.9 ± 1.0 ***	2.0 ± 0.7 ***	2.1 ± 0.5 ***	1.9 ± 0.7 ***
5-LO	LTB4	66.3 ± 30.3	90.9 ± 32.0	100 ± 39	50.1 ± 18.4	58.8 ± 22.9	13.1 ± 3.3 ***
	t-LTB4	23.8 ± 9.0	35.2 ± 9.8	38.6 ± 13.6	19.4 ± 6.0	20.5 ± 8.0	3.9 ± 1.2 ***
	20-OH-LTB4	4.2 ± 2.6	3.5 ± 1.8 *	4.1 ± 2.0	3.2 ± 1.7	2.5 ± 1.4 *	0.3 ± 0.1 ***
	5-HETE	145 ± 50	224 ± 66	244 ± 81	107 ± 29	137 ± 48	23.5 ± 3.9 ***
	5,15-diHETE	110 ± 48	52.7 ± 5.6	46.1 ± 5.6 *	40.1 ± 4.7 **	46.7 ± 8.7 *	118 ± 78
	5S,6R-diHETE	5.2 ± 2.2	7.7 ± 2.4	7.4 ± 2.4	3.5 ± 0.9	4.4 ± 1.9	0.5 ± 0.2 ***
	5-HEPE	3.6 ± 0.8	4.8 ± 1.2	6.0 ± 1.7	2.3 ± 0.6	3.4 ± 0.8	0.8 ± 0.0 ***
12/15-LO	12-HETE	2242 ± 893	2487 ± 1119	2168 ± 989	1991 ± 932	1864 ± 780	2178 ± 909
	15-HETE	132 ± 44	124 ± 53	68.4 ± 28.8 *	36.2 ± 13.4 ***	29.3 ± 7.9 ***	34.1 ± 10.2 ***
	12-HEPE	108 ± 40	107 ± 48	106 ± 50	95.8 ± 45.3	80.0 ± 31.6 *	83.6 ± 32.5 *
	14-HDHA	75.5 ± 16.6	70.3 ± 20.0	70.7 ± 19.1	63.0 ± 15.0	70.4 ± 18.0	66.2 ± 15.5
	14-HDPA	87.6 ± 24.9	89.2 ± 29.2	87.8 ± 23.8	67.0 ± 19.2 *	85.1 ± 21.5	86.4 ± 24.4
	RvD6	4.0 ± 1.4	3.7 ± 2.0	2.0 ± 0.8	1.7 ± 0.8 ***	1.6 ± 0.8 ***	1.4 ± 0.3 **
others	9-HODE	117 ± 19	106 ± 23	101 ± 23	98.6 ± 22.3	108 ± 22	87.2 ± 3.6
	13-HODE	163 ± 30	149 ± 38	147 ± 40	153 ± 38	165 ± 35	118 ± 8
	4-HDHA	3.5 ± 0.4	3.2 ± 0.6	4.3 ± 0.5	3.7 ± 0.3	3.8 ± 0.5	3.4 ± 0.6
	13-HDHA	10.3 ± 2.5	8.5 ± 3.4	6.2 ± 2.2	4.5 ± 1.7 ***	5.0 ± 1.3 *	4.9 ± 1.0 *
	13-HDPA	18.9 ± 4.6	17.4 ± 6.0	15.8 ± 4.3 *	16.6 ± 4.5	18.1 ± 4.5	16.3 ± 3.6
free PUFA	AA	3252 ± 695	3916 ± 1110	4581 ± 1332	4295 ± 1144	3863 ± 905	3857 ± 874
	EPA	894 ± 280	695 ± 168	1217 ± 677	1169 ± 558	979 ± 334	625 ± 67
	DHA	1663 ± 266	1535 ± 191	2011 ± 411	1869 ± 334	1714 ± 309	1543 ± 221

Figure S12. Effect of compound 2 on the lipid mediator profile of LPS-primed monocytes. After pre-stimulation with LPS to induce COX-2 expression, monocytes were treated with vehicle (DMSO), compound **2**, indomethacin, or celecoxib and activated with A23187. Lipid mediator profiles were analyzed by UPLC-MS/MS. The color scale indicates the percentage changes of lipid mediators vs. vehicle. The values represent mean ± standard error of the mean (s.e.m.) from n = 3 independent experiments in pg. *p < 0.05, **p < 0.01, ***p < 0.001 vs. vehicle; repeated measures two-way ANOVA + Tukey's *post hoc* test of logarithmized data. (di)HETE, (di)hydroxy-eicosatetraenoic acid; HEPE, hydroxy-eicosapentaenoic acid; LT, leukotriene; t-LTB_4, trans-LTB_4 isomers; 20-OH-LTB_4, 20-hydroxy-LTB_4; HDHA, hydroxy-docosahexaenoic acid; Rv, resolvin; HODE, hydroxy-octadecadienoic acid; HDPA, hydroxy-docosapentaenoic acid; AA, arachidonic acid; EPA, eicosapentaenoic acid; DHA, docosahexaenoic acid.

Table S4. X-ray data collection and refinement statistics

**Data collection statistics**	
Wavelength (Å)	0.9795
Space group	$P6_5$
Unit cell dimensions (Å)	a = 181.6, b= 181.6, c= 103.4
Angles (°)	α=β= 90, γ=120
Resolution range (Å)	50 – 3.35
Total observations	358,787
Unique observations	14,537
Completeness (%)	97.8 (96.9)
R_{sym} (%)	12.3(84.4)
R_{pim} (%)	6.9 (43.6)
CC1/2	0.96 (0.33)
$\langle I\rangle/\langle\sigma(I)\rangle$	31.7 (2.3)
**Refinement statistics**	
PDB-ID	7JXT
Resolution (Å)	14.99 – 3.35
Number of reflections [a]	23,088
R_{work}/R_{free} [b] (%)	20.97 / 24.99
Copies in Asymmetric Unit	2
Num. water molecules	0
MolProbity ClashScore	4.62
R.M.S.D. from ideal length (Å)/angles (°)	0.003 / 0.63
Ramachandran (%) favor/allow/disallow	96.6 / 3.4 / 0.0

In parenthesis are statistics for the outer resolution shell (3.47-3.35 Å).
[a] Number of reflections with |Fobs|/sigma|Fobs| >2.35 used for refinement
[b] R_{free} was calculated using 5% of randomly chosen reflections.

^{1}H and ^{13}C NMR spectra of synthesized compounds

^{1}H NMR of **intermediate 1** in acetone-d_6:

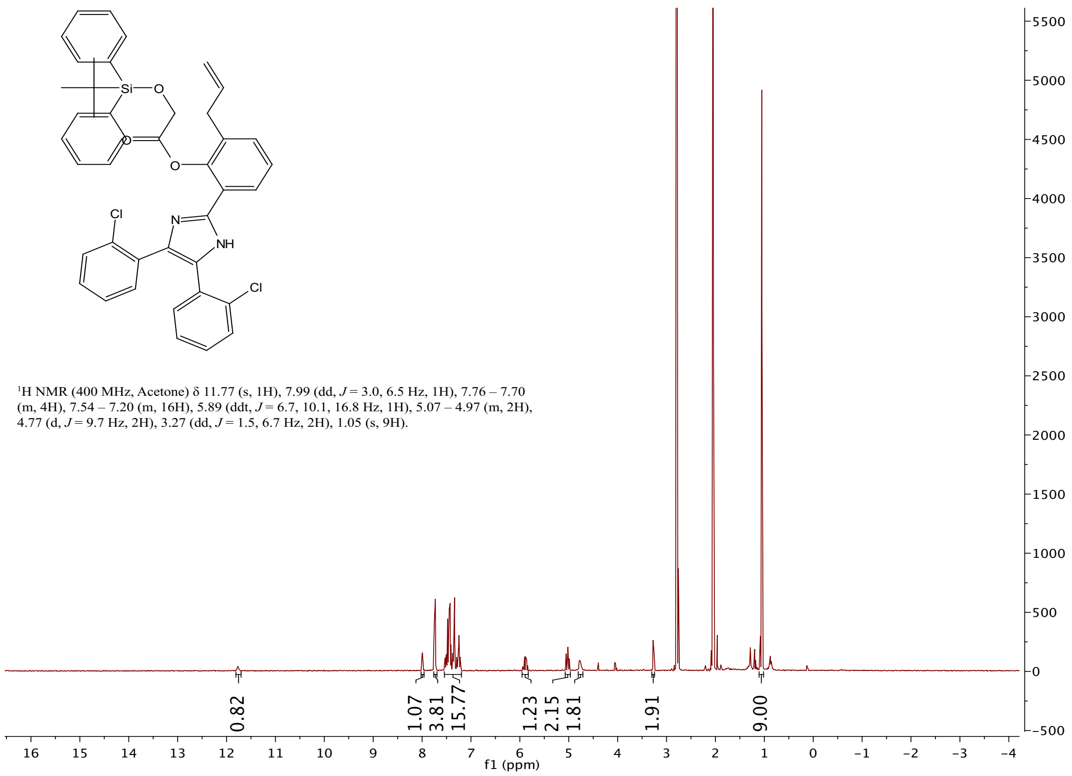

^{1}H NMR (400 MHz, Acetone) δ 11.77 (s, 1H), 7.99 (dd, J = 3.0, 6.5 Hz, 1H), 7.76 – 7.70 (m, 4H), 7.54 – 7.20 (m, 16H), 5.89 (ddt, J = 6.7, 10.1, 16.8 Hz, 1H), 5.07 – 4.97 (m, 2H), 4.77 (d, J = 9.7 Hz, 2H), 3.27 (dd, J = 1.5, 6.7 Hz, 2H), 1.05 (s, 9H).

^{13}C NMR of **intermediate 1** in DMSO-d_6:

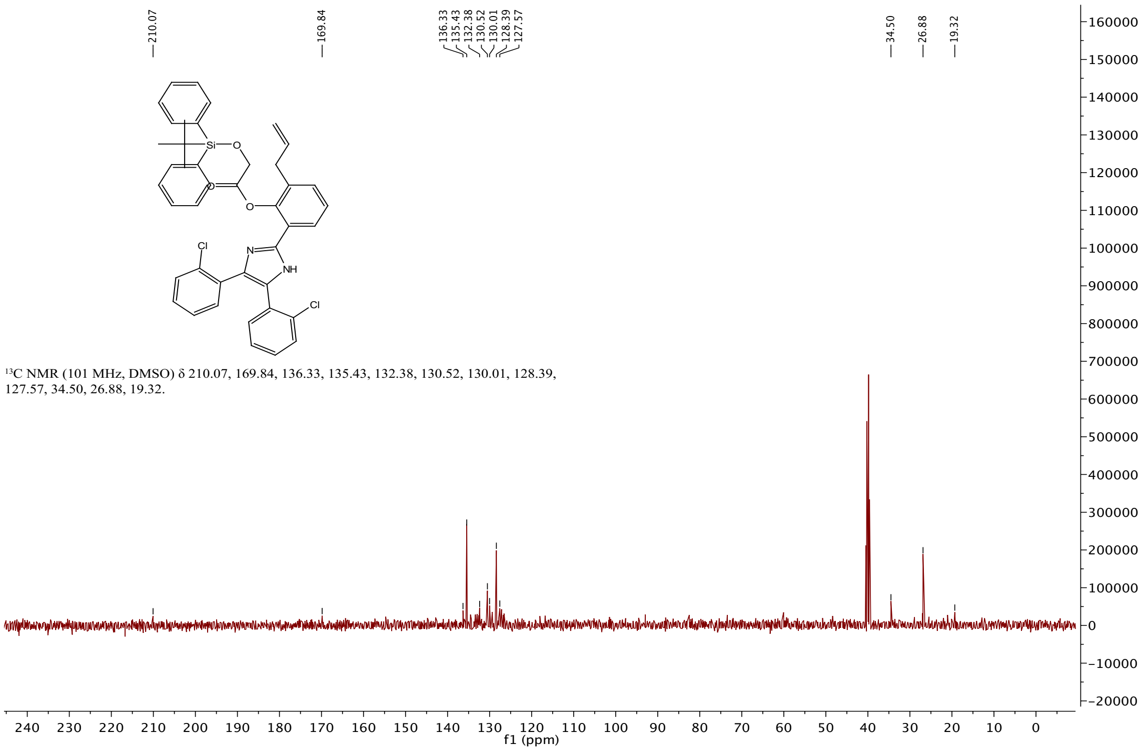

^{13}C NMR (101 MHz, DMSO) δ 210.07, 169.84, 136.33, 135.43, 132.38, 130.52, 130.01, 128.39, 127.57, 34.50, 26.88, 19.32.

^{1}H NMR of compound **2** in DMSO-d_6:

^{1}H NMR (500 MHz, DMSO) δ 12.88 – 12.81 (m, 1H), 7.90 (ddt, *J* = 1.9, 4.2, 5.8 Hz, 1H), 7.53 (d, *J* = 8.0 Hz, 1H), 7.47 (ddd, *J* = 1.8, 3.8, 7.4 Hz, 1H), 7.40 – 7.29 (m, 7H), 7.29 – 7.24 (m, 1H), 5.97 – 5.86 (m, 1H), 5.52 (dq, *J* = 2.0, 4.3 Hz, 1H), 5.13 (dt, *J* = 2.1, 17.2 Hz, 1H), 5.10 – 5.05 (m, 1H), 4.41 (q, *J* = 3.5, 4.5 Hz, 2H), 3.35 – 3.30 (m, 2H).

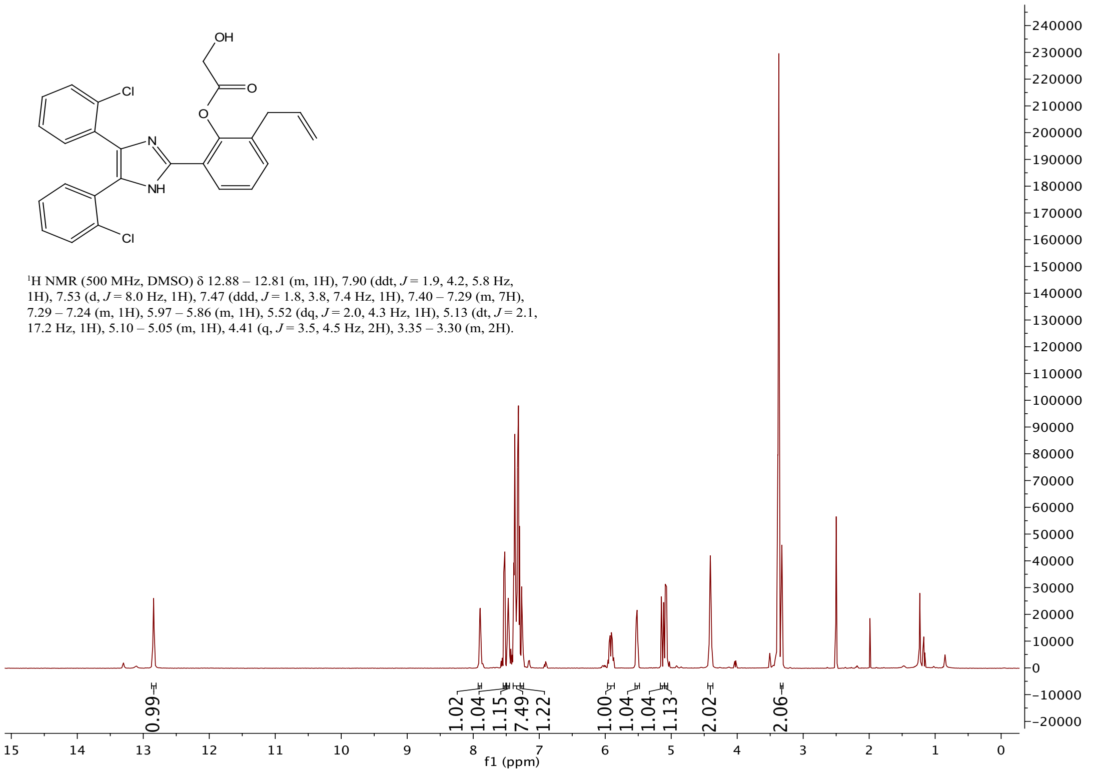

^{13}C NMR of compound **2** in DMSO-d_6:

^{13}C NMR (126 MHz, DMSO) δ 171.97, 145.74, 143.00, 137.62, 136.43, 134.55, 134.40, 133.30, 132.61, 132.53, 130.65, 130.60, 130.41, 130.22, 129.98, 129.44, 127.57, 127.54, 127.38, 127.31, 126.88, 126.64, 123.65, 117.02, 60.73, 34.53.

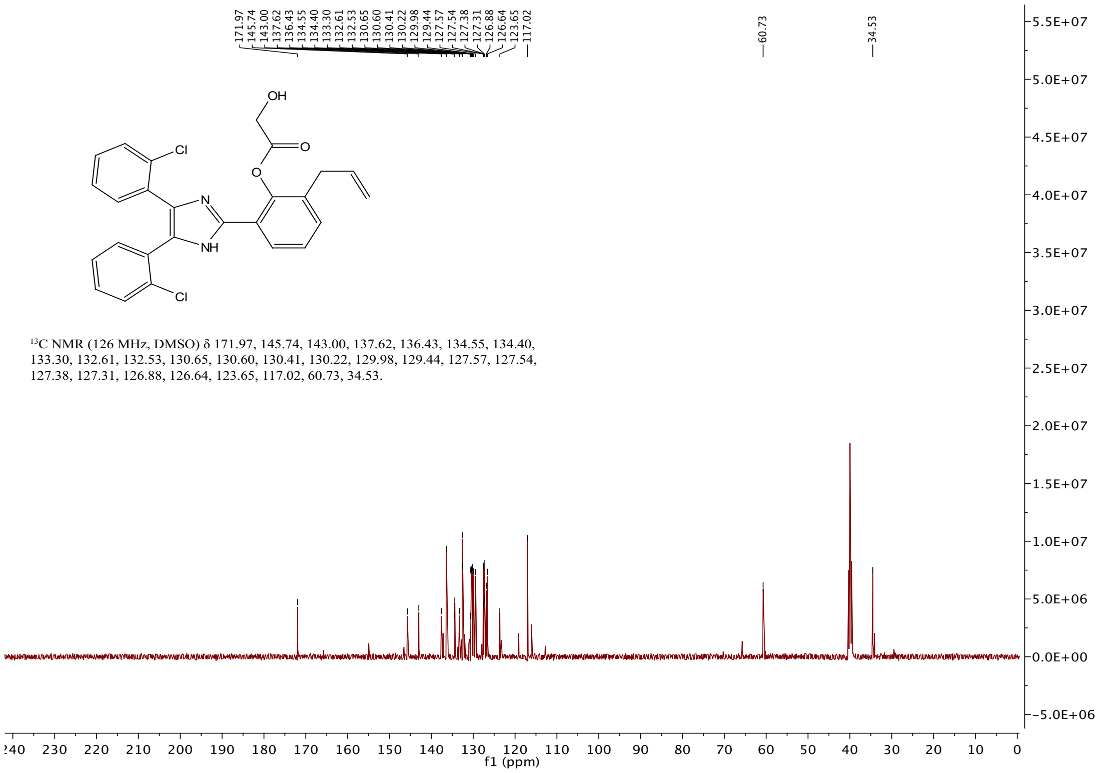

^{1}H NMR of compound **2a** in DMSO-d_6:

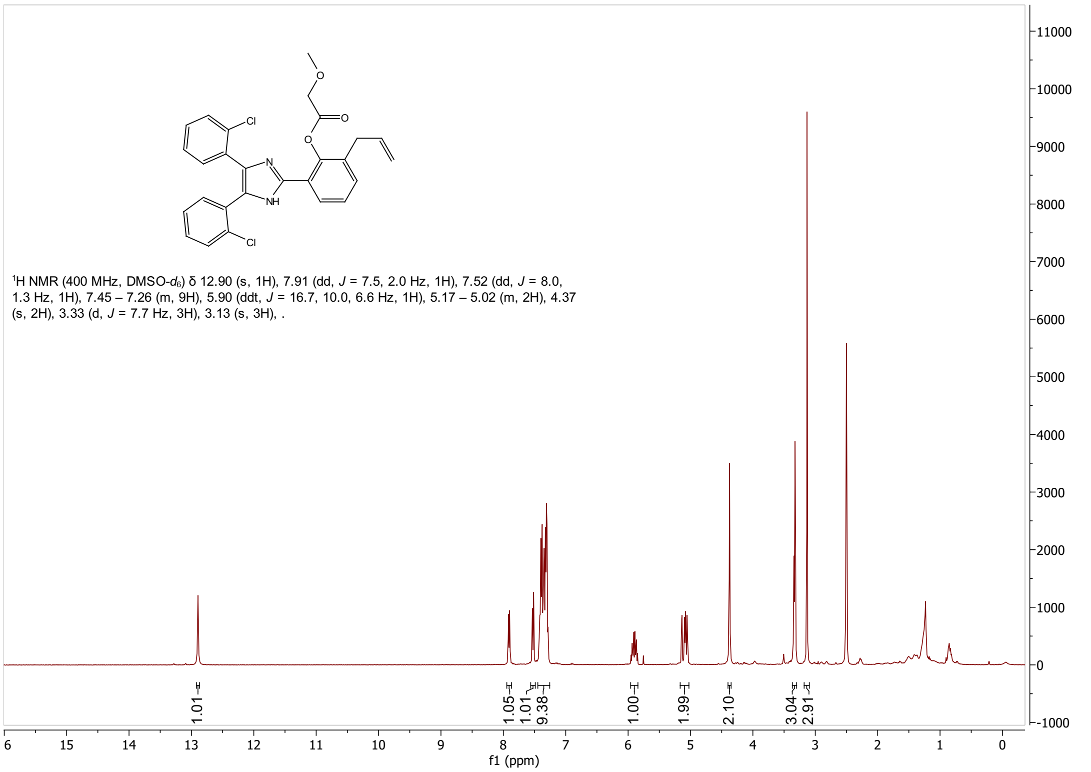

^{13}C NMR of compound **2a** in DMSO-d_6:

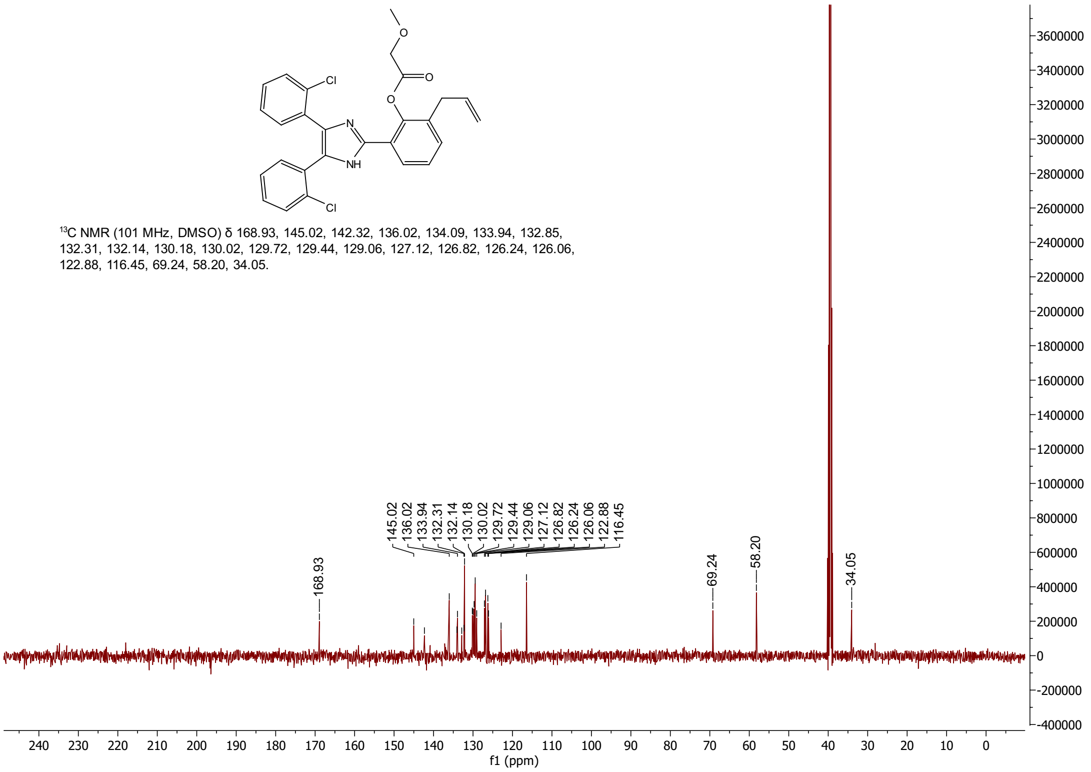

^{1}H NMR of compound **2b** in DMSO-d_6:

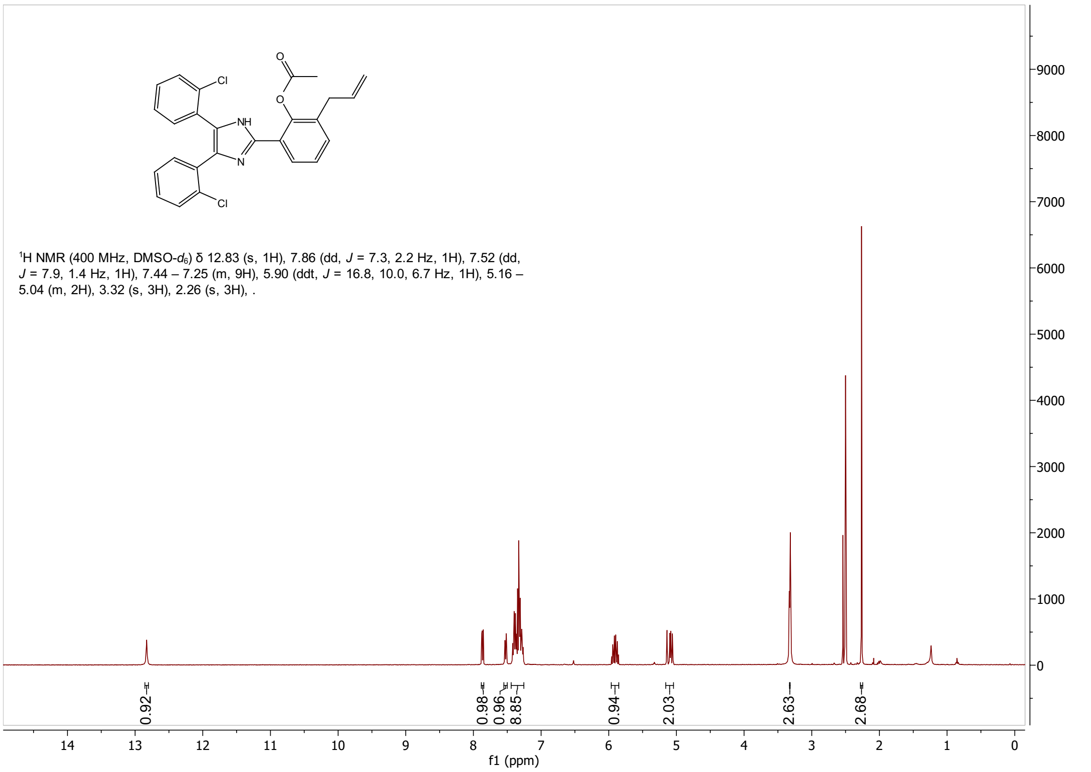

^{13}C NMR of compound **2b** in DMSO-d_6:

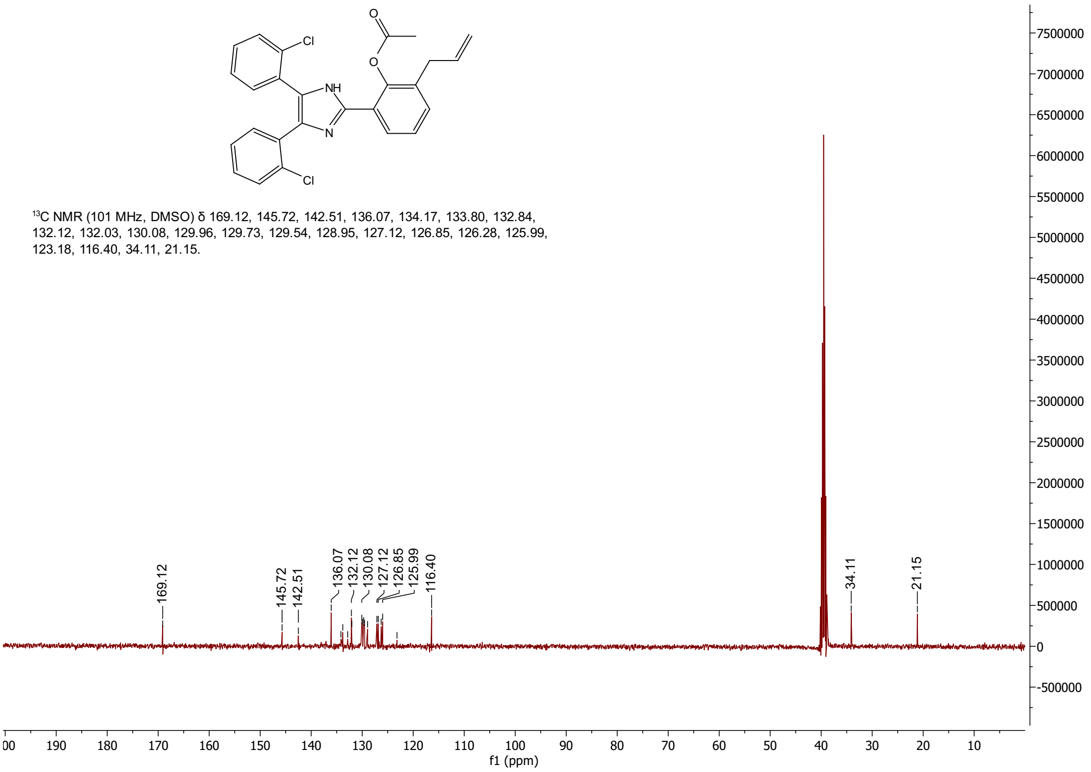

^{1}H NMR of compound **3** in DMSO-d_6:

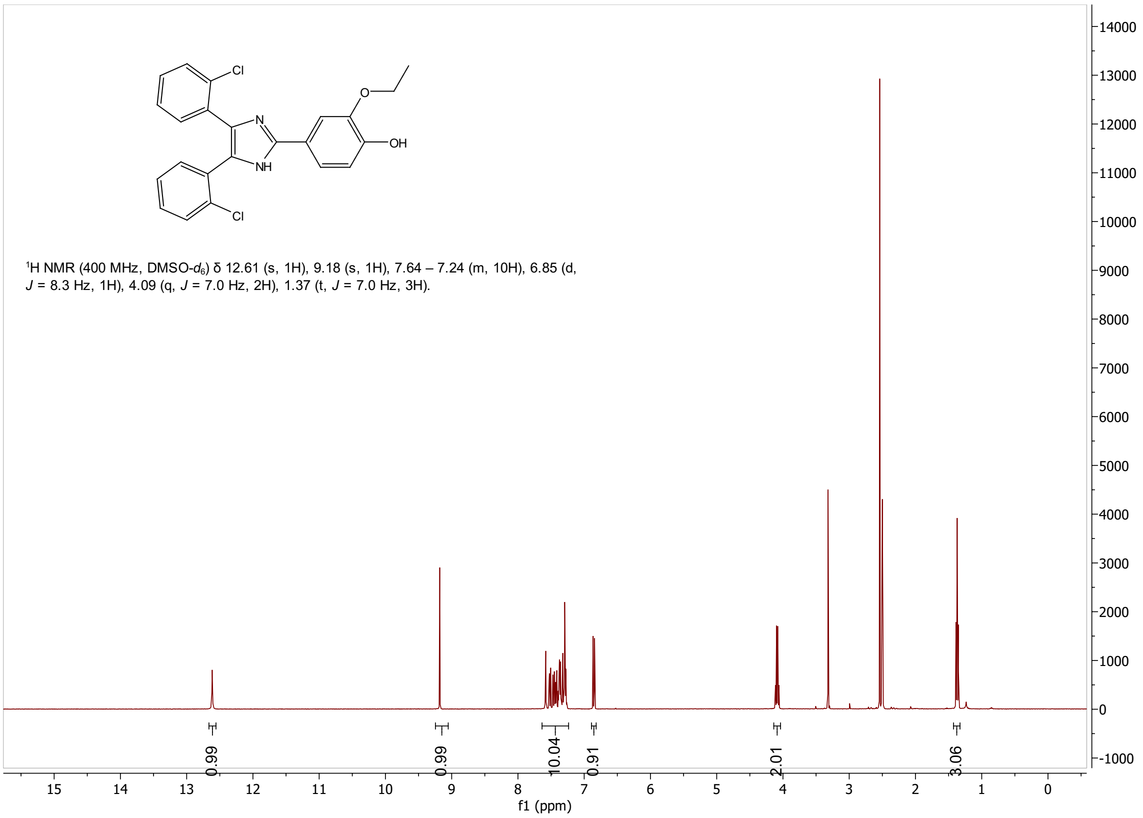

^{13}C NMR of compound **3** in DMSO-d_6:

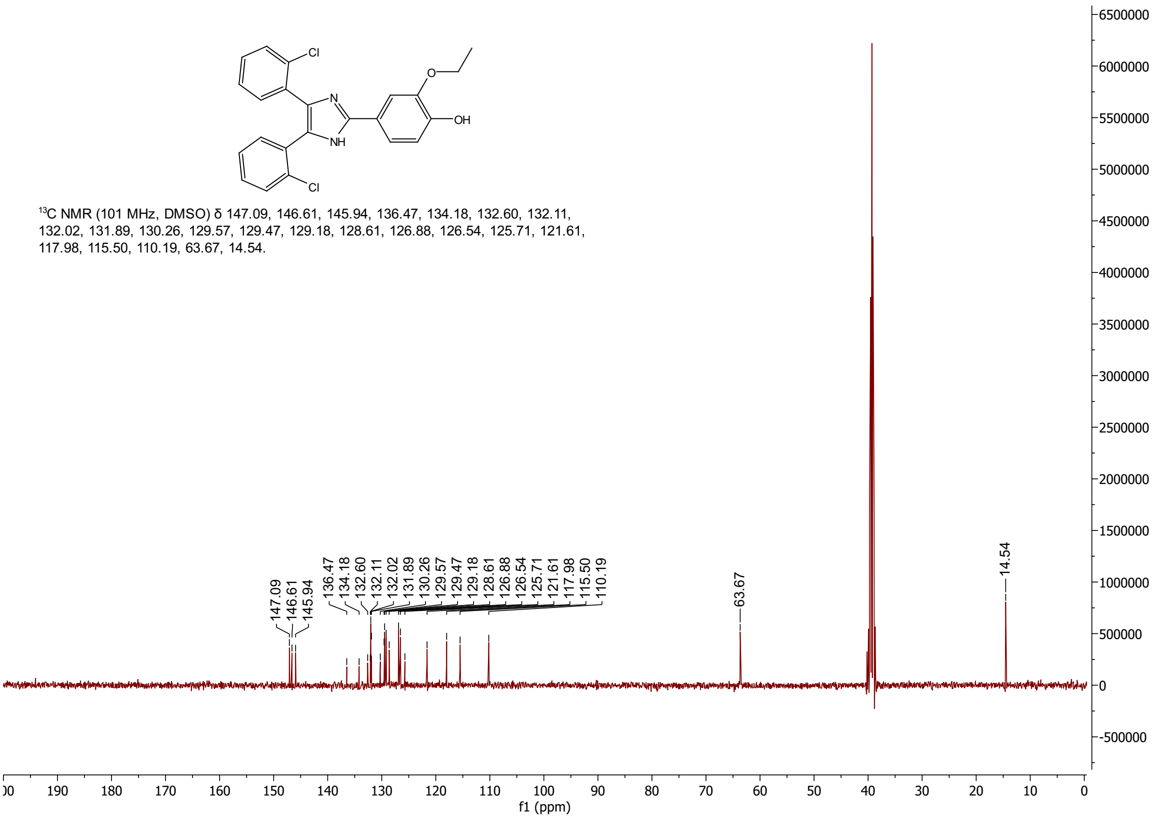

^{1}H NMR of compound **4** in DMSO-*d₆*:

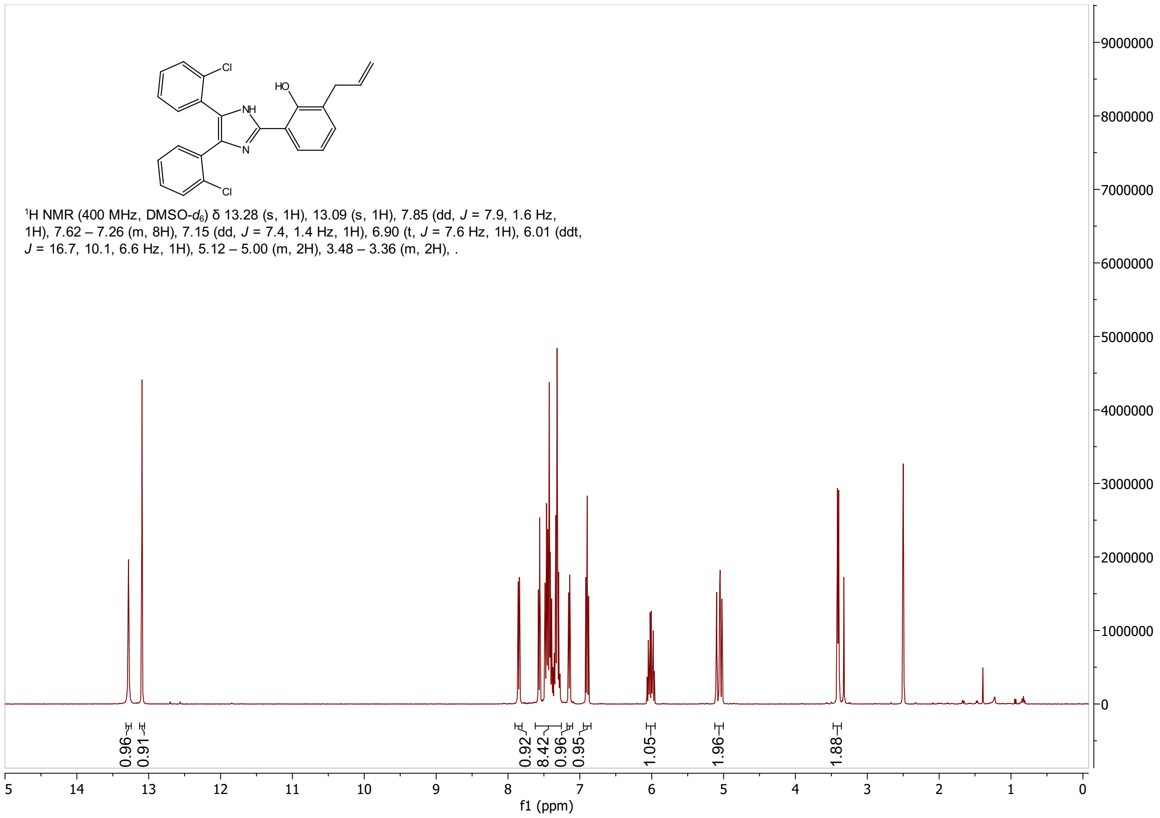

^{13}C NMR of compound **4** in DMSO-*d₆*:

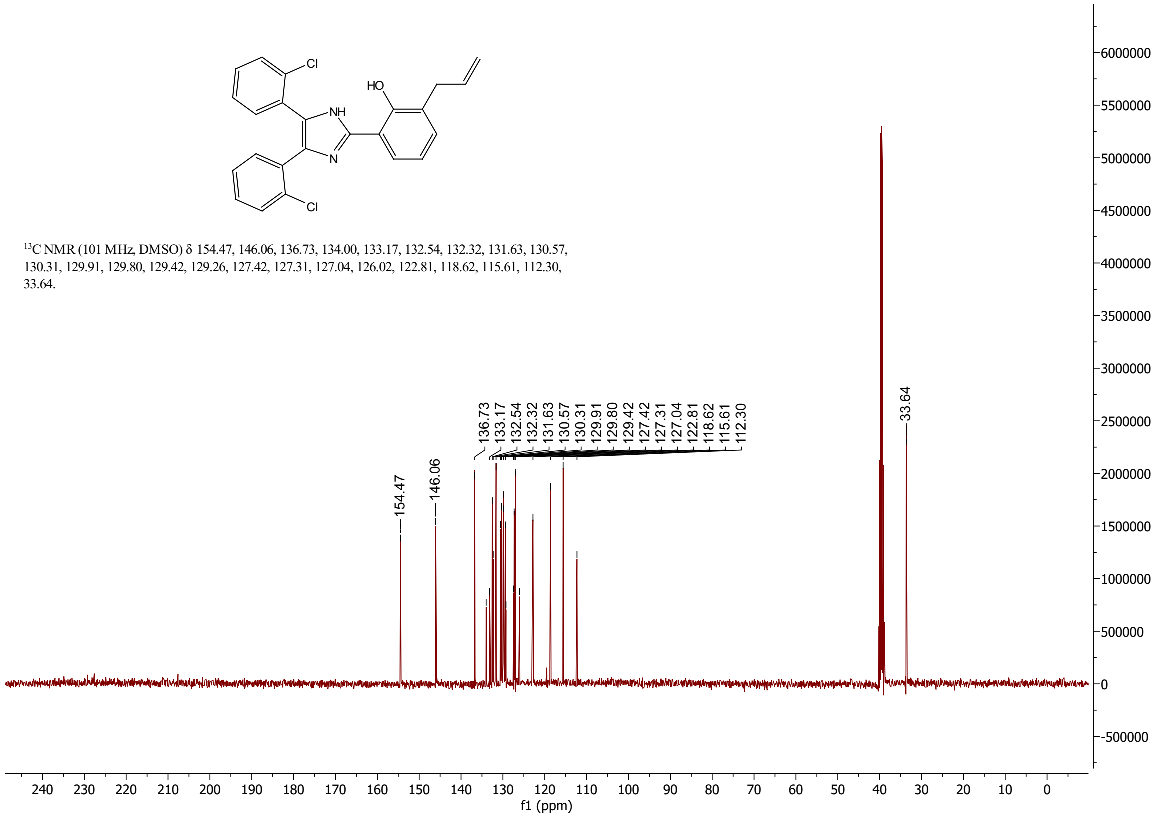

Infrared (IR) Spectra

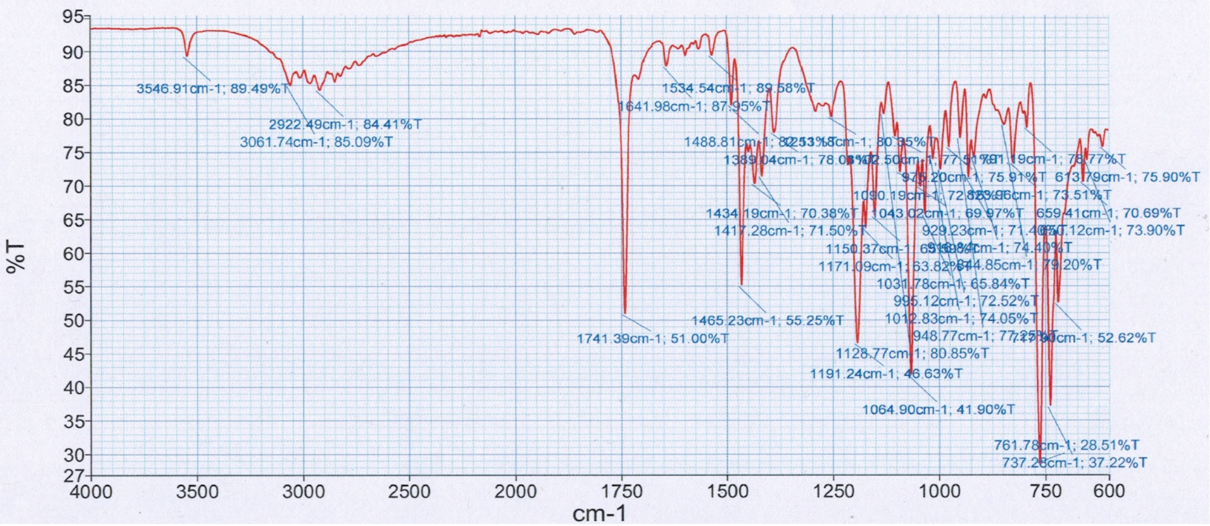

IR spectrum of compound **2**

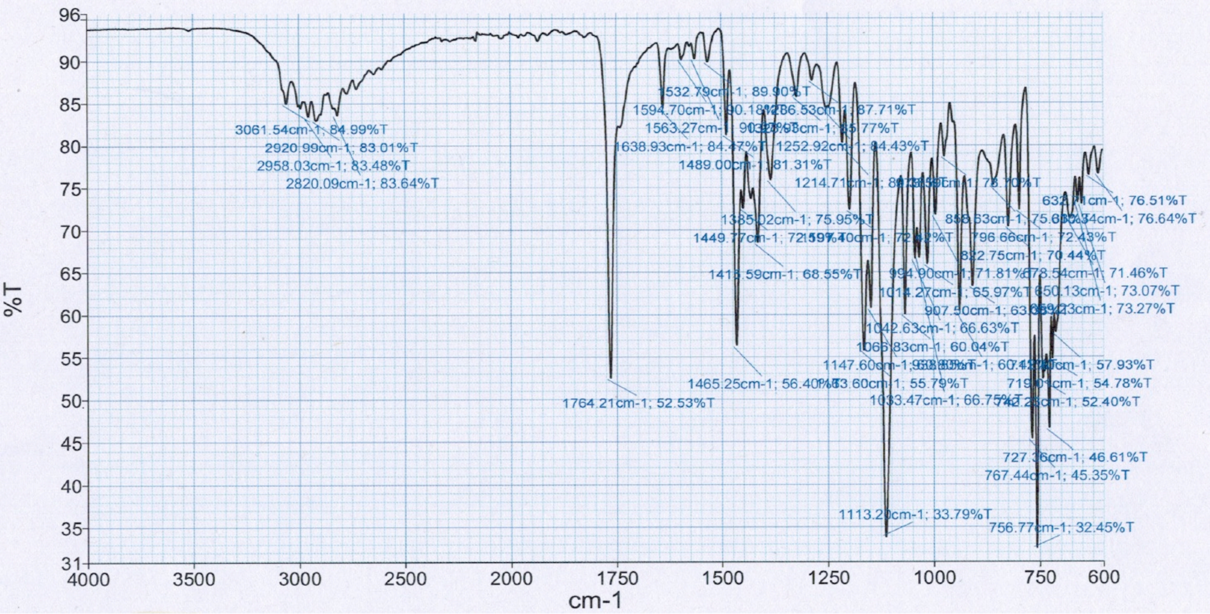

IR spectrum of compound **2a**

Ultraviolet–visible (UV/Vis) Spectra

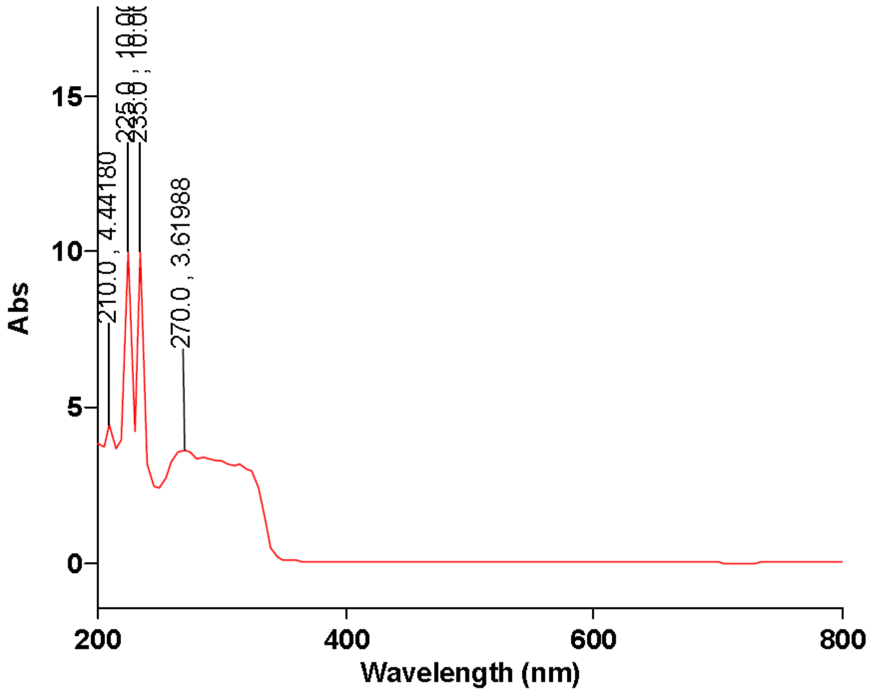

UV/Vis spectrum of compound **2**

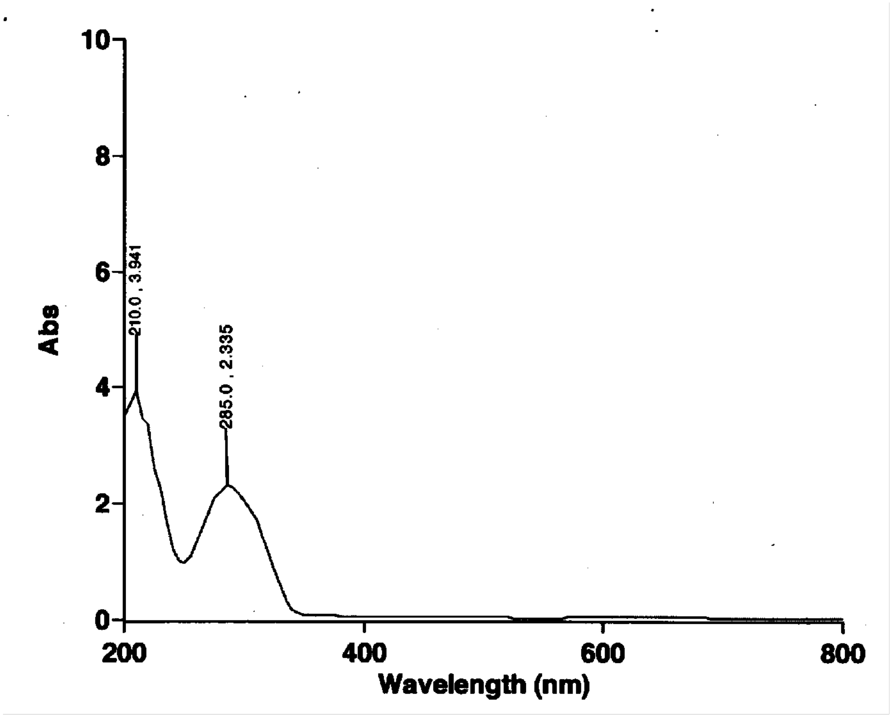

UV/Vis spectrum of compound **2a**
